# *In vitro* safety evaluation of dopamine D_3_R antagonist, *R*-VK4-116, as a potential medication for the treatment of opioid use disorder

**DOI:** 10.1371/journal.pone.0315569

**Published:** 2024-12-16

**Authors:** Rahul M. Nandre, Amy Hauck Newman, Pramod S. Terse

**Affiliations:** 1 Therapeutic Development Branch, Division of Preclinical Innovation, National Center for Advancing Translational Sciences, National Institute of Health, Rockville, Maryland, United States of America; 2 Medicinal Chemistry Section, Molecular Targets and Medications Discovery Branch, National Institute on Drug Abuse–Intramural Research Program, National Institute of Health, Baltimore, Maryland, United States of America; University of Nis Faculty of Medicine: Univerzitet u Nisu Medicinski Fakultet, SERBIA

## Abstract

*R*-VK4-116 is currently being developed as a medication to treat opioid use disorder (OUD). To characterize *in vitro* safety properties of *R-*VK4-116, metabolic stability in hepatocytes or liver microsomes, metabolite identification, metabolism/transporter-mediated drug interactions, lysosomal perturbation, mitochondrial toxicity, off-target enzyme effects, cellular and nuclear receptor functional assays, electrophysiological assays, CiPA, KINOME*scan*^TM^, plasma protein binding, phospholipidosis and steatosis assays were performed. Overall, *R-*VK4-116 was metabolically stable in hepatocytes and microsomes. Four major metabolites were detected: two mono-oxidation products, one di-oxidation product, and one demethylated plus di-oxidation product. CYP2D6 and CYP3A4 were major contributors in *R-*VK4-116 metabolism. Further, *R-*VK4-116 did not induce/inhibit CYP enzymes. However, *R-*VK4-116 inhibited BCRP/P-gp, and showed antagonist effects on α1A*(h)*, H1*(h)* and agonist effect on hGABAA∞1β2γ2 at 10 μM. *R-*VK4-116 inhibited hERG and Kir2.1 at a high concentration of 100 μM. KINOME*scan*^TM^ provided 5 hits (CHEK2, HPK1, MARK3, SRPK2 and TNK1) with Kds of >10 μM. Further, *R-*VK4-116 was bound to human, rat and dog plasma proteins (~83–93%). *R-*VK4-116 did not induce lysosome perturbation, mitochondrial toxicity, phospholipidosis, or steatosis at ≤10 μM. These results demonstrated that *R-*VK4-116 possesses favorable *in vitro* safety properties and supports further development as a potential medication for OUD.

## Introduction

Drug overdose deaths, largely fueled by the synthetic opioid fentanyl, continue to escalate in the United States, with >100,000 fatalities per year since 2021 (https://nida.nih.gov/research-topics/trends-statistics/overdose-death-rates). To address the opioid crisis, the National Institute on Drug Abuse (NIDA) proposed a list of highest priority pharmacological targets for medication development [1]. Among these, the dopamine D_3_R (D_3_R) was one of the highlighted targets [2]. Although D_3_R has been a target for the development of medications to treat various substance use disorders, poor absorption, distribution, metabolism and excretion (ADME), or cardiotoxicity in experimental animals of earlier developed D_3_R antagonist, especially in the presence of cocaine, prevented successful clinical transition [3, 4].

*R-*VK4-116 (*N*-(4-(4-(3-chloro-5-ethyl-2-methoxyphenyl)piperazin-1-yl)-3-hydroxybutyl)-1H-indole-2-carboxamide) was first reported in 2016 as a highly selective D_3_R antagonist [5]. *R-*VK4-116 (5, 15 and 25 mg/kg) decreased oxycodone self-administration, suppressed oxycodone-enhanced dopamine-dependent optical brain stimulation reward, dose-dependently diminished conditioned place aversion induced by naloxone-precipitated opioid withdrawal, as well as enhancing oxycodone-induced analgesia in rodents [6]. It was also effective in reducing dose escalation of oxycodone as well as reducing withdrawal-associated hyperalgesia in both male and female rats at 5, 15 and 25 mg/kg [7] supporting its further development. Resolution of the enantiomers of (±)VK4-116, demonstrated *R-*VK4-116 to be the eutomer at D_3_R [8]; this compound (up to 25 mg/kg) did not potentiate the cardiovascular effects of either cocaine or oxycodone in rats [9].

In order to further consider *R-*VK4-116 as a potential clinical candidate, we evaluated its metabolic stability in hepatocytes or liver microsomes, and metabolites were identified. Further, metabolism and transporter-mediated drug interaction assays were performed. In addition, *R-*VK4-116 was evaluated for lysosomal perturbation, mitochondrial toxicity, oxidative stress and apoptosis potential in HepG2, off-target enzyme effects, cellular and nuclear receptor functional assays, electrophysiological assays, CiPA, KINOME^TM^ profiling, plasma protein binding, phospholipidosis and steatosis. The results of *in vitro* exploratory studies suggest a low potential of *R-*VK4-116 to cause off-target toxicity.

## Materials and methods

### Test article and cells

*R-*VK4-116 (lot no. TRND00600611-03; synthesized with purity 99.16% at NCATS, NIH, and lot no. 14853-C-R0-01-55-01; synthesized with purity 97.9% at Curia, NY) was prepared as a 10 mM stock in DMSO and stored at -20°C. The stock of TRND00600611-03 (*R-*VK4-116-2HCl) was diluted appropriately for all assays except CYP phenotyping and SLC transporter mediated assays. Lot number 14853-C-R0-01-55-01 (*R-*VK4-116-HCl) stock was diluted appropriately to conduct CYP phenotyping and SLC transporter mediated assays. CryostaX® pooled mixed gender human (5 male and 5 female) hepatocytes, Male Sprague Dawley (Total 7) rat hepatocytes, Male Beagle dog (Total 3) hepatocytes (XenoTech, LLC, Kansas City, KS) were used for cell-based assays. All assays were repeated.

### Metabolic stability in hepatocyte

To evaluate metabolic stability, *R-*VK4-116 (0.5 and 10 μM) was incubated in duplicate with human, rat and dog hepatocytes (1 x 10^6^ cells/ml of OptiIncubate media) in suspension, respectively (n = 2 at each time point). *R-*VK4-116 was also incubated in the absence of hepatocytes (n = 6 at each time point) to give information about stability and non-specific binding during the experiment (Table A in S1 File). Reactions were terminated by removing 50 μl aliquots at selected time points (0, 30, 60, 90 and 120 min) and mixing with 200 μl of acetonitrile (Mallinckrodt Baker, Inc, Phillipsburg, NJ) containing ethyl nicotinate (Sigma-Aldrich, St Louis, MO) as internal standard. CYP3A4 substrate Midazolam (10 μM) was prepared and incubated in the same manner. Following brief centrifugation, the supernatants were further diluted (10- and 100-fold for 0.5 μM and 10 μM *R-*VK4-116 samples (n = 2 at each time point), respectively; 100-fold for 10 μM midazolam samples (n = 6 at each time point)) with acetonitrile:water:formic acid (25:75:0.2, v/v/v), and analyzed by LC-MS/MS. In addition, *in vitro* half-life (t_1/2_, min) and intrinsic clearance (CL_int_, μl/min/10^6^ cells) were calculated [10, 11].

### Metabolic stability and metabolite identification in liver microsomes

To evaluate metabolic stability, *R-*VK4-116 (1 and 10 μM) was incubated in duplicate with human, rat, dog, or monkey liver microsomes (0.5 mg/ml) (XenoTech, LLC, Kansas City, KS) (n = 2 at each time point) and appropriate cofactors (2.5 mM NADPH and 3.3 mM MgCl_2_) in 100 mM phosphate buffer, pH 7.4, in a 37°C water bath. Reactions were stopped by removing 100 μl aliquots at selected time points (0, 30, 60, 90 and 120 min) and mixing with 200 μl of acetonitrile containing ethyl nicotinate as internal standard. Following brief centrifugation, the 1 and 10 μM *R-*VK4-116 samples were diluted 10- and 100-fold, respectively, then analyzed by LC-MS/MS. Appropriate controls were included such as incubation of 1 and 10 μM *R-*VK4-116 with heat-inactivated (HI) microsomes (0.5 mg protein/ml) for 0 and 120 min (n = 2 at each time point), and incubation of 10 μM midazolam with all incubation components (0, 30, 60, 90 and 120 min) or with HI microsomes for 0 and 120 min (n = 2 at each time point).

To detect major metabolites, microsomal samples were prepared with incubation of 10 μM *R-*VK4-116 with the same incubation conditions as for the metabolic stability analysis. Reactions were stopped by removing 100 μl aliquots at 120 min and mixing with 200 μl of acetonitrile. Following brief vortexing and centrifugation, the supernatant was transferred to an HPLC vial fitted with a 300 μl glass insert for metabolite analysis using a Sciex 4000QTrap mass spectrometer. Appropriate controls were included such as incubation of all reaction components except *R-*VK4-116 and incubation of 10 μM *R-*VK4-116 with either HI microsomes or lacking NADPH at 0 and 120 min.

### CYP phenotyping

CYP phenotyping involved the identification of CYP isoforms, which involved in the metabolism of *R-*VK4-116 using recombinant CYP1A2, CYP2B6, CYP2C8, CYP2C9, CYP2C19, CYP2D6, and CYP3A4 (XenoTech, LLC, Kansas City, KS), followed by determination of the K_m_ and V_max_ for the metabolites formed by the major CYP isoforms [12]. *R-*VK4-116 (1 μM) was incubated in duplicate with each CYP isoform individually, control bactosomes (XenoTech, LLC, Kansas City, KS), or human liver microsomes (200-donor mixed gender; XenoTech, LLC, Kansas City, KS), and cofactors (2.5 mM NADPH and 3.3 mM MgCl_2_) in 0.1 M phosphate buffer, pH 7.4, in a 37°C water bath. In addition, *R-*VK4-116 was also incubated with all components in the absence of NADPH. Aliquots (100 μl) were removed at 0, 15, 30, 60, 90, and 120 min, and mixed with 200 μl of acetonitrile containing ethyl nicotinate as internal standard (IS). Following brief vortexing and centrifugation, 50 μl of supernatant was mixed with 450 μl of 25:75:0.2 acetonitrile/water/formic acid for analysis by LC-MS/MS to determine the remaining *R-*VK4-116 at each time point based on peak area ratio (PAR) of *R-*VK4-116 to IS. As a positive control, the known substrate of each individual CYP isoform (phenacetin for CYP1A2, bupropion for CYP2B6, paclitaxel for CYP2C8, diclofenac for CYP2C9, diazepam for CYP2C19, bufuralol for CYP2D6, and testosterone for CYP3A4) was also incubated.

### CYP induction and inhibition

An upfront and concurrent test to assess the toxicity potential of the *R-*VK4-116 to human hepatocytes was conducted using the 3-[4, 5-Dimethyl-2-thiazolyl]-2,5-diphenyl-2H-tetrazolium bromide (MTT) assay. The subsequent study was designed to assess the potential of *R-*VK4-116 (0.01, 0.1, 1, 3, 5 and 10 μM) to induce CYP isoforms 1A2, 2B6 and 3A4 mRNA and *in situ* catalytic activity using primary cultured human hepatocytes with 48-hr incubation [12–15]. Rifampicin as a positive control and solvent (0.1% DMSO) vehicle control were also included. The acceptance criteria for CYP induction was ≥ 2-fold induction for significant CYP activity, while the acceptance criteria for CYP mRNA was ≥ 4-fold for significant CYP induction.

To determine the potential for CYP450 inhibition, *R-*VK4-116 was incubated at 0.1, 0.3, 1, 3, 10, 30, 60 and 100 μM with human liver microsomes, cofactors, and specific CYP probe substrates for 20 min. Positive control compounds that are known to inhibit specific CYP isoforms were also included and incubated in the same manner as *R-*VK4-116. Samples were analyzed by LC-MS/MS. The percent CYP activity in the test article or specific inhibitor samples relative to the control samples (not containing *R-*VK4-116/inhibitors) was calculated as follows:

$$\%\;CYP\;activity=\left(\frac{Substrate\;metabolite\;response,PAR\;in\;the\;presence\;of\;inhibitor\;or\;test\;article}{\;Substrate\;metabolite\;mean\;PAR\;in\;Control}\right)\;X\;100$$

If CYP activity was less than 70% of the control, the significant inhibition of CYP enzyme activity in the presence of *R-*VK4-116 was considered.

### Transporter mediated assay

**Fig 1:**
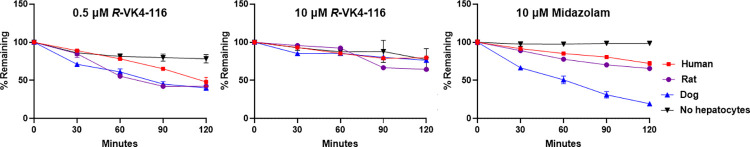
Metabolism of *R-*VK4-116 and positive control, midazolam using human, rat, and dog hepatocytes and in the absence of hepatocytes (% remaining Vs minutes; mean).

To assess the potential for *R-*VK4-116 to be a substrate or inhibitor of human ABC (BCRP, P-gp) and SLC (OAT1, OAT3, OCT2, OATP1B1, OATP1B3, MATE1, and MATE2-K) mediated transporters, the uptake test system for each transporter was a polarized monolayer of MDCK-II cells grown on permeable supports [12]. The MDCK-II cells were treated to express the transporter of interest. The transport of each probe substrate was determined by radiometric detection (Fig 1 in S1 File). *R-*VK4-116 was quantified by LC/MS/MS. For the uptake assay, the net transporter-mediated uptake-rate (V) of the substrate by each SLC transporter was calculated as follows:

$$Transporter\;–mediated\;update\;rate\;(pmol/min/{cm}^{2})=\frac{\left(Cellular\;accumulation\;in\;cells\;expressing\;the\;transporter)-(Mean\;cellular\;accumulation\;in\;control\;cells\right)}{Surface\;area\times incubation\;time}$$

Fold of activity and percent inhibition were determined as follows:

$$Fold\;of\;activity=\frac{{\left(Mean\;uptake\;rate\right)}_{transporter\;expressing\;cells}\;}{{\left(Mean\;uptake\;rate\right)}_{control\;cells}}$$

$$Percent\;inhibition=100-100\times \frac{\;{\left(Transport‐mediated\;update\;rate\right)}_{with\;inhibitor}}{{\left(Transport‐mediated\;update\;rate\right)}_{vehicle\;control}}$$

For BCRP and P-gp, the apparent permeability Papp and Efflux Ratio (ER) were calculated [16]. Net basal (B) to apical (A) flux (B → A) of the substrate transport (pmol/min/cm^2^) by BCRP or P-gp was calculated by subtracting A → B flux from B → A flux.

Percent inhibition was calculated by dividing the ER in the presence of *R-*VK4-116 or the reference inhibitor by the ER in the absence of the inhibitor:

$$Percent\;inhibition=100-\left[100\times (\frac{{\left(ER-1\right)}_{with\;inhibitor}}{{\left(mean\;ER-1\right)}_{without\;inhibitor}})\right]$$

### Lysosomal trapping assay

**Fig 2:**
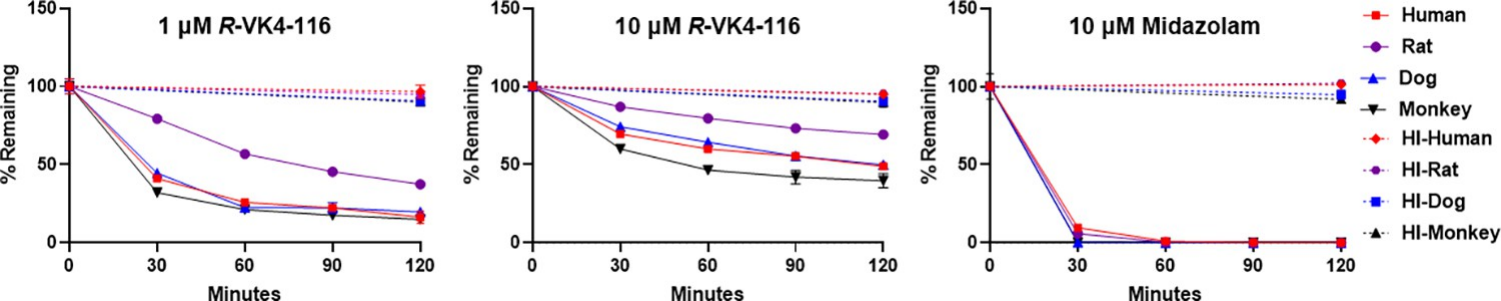
Metabolic stability of *R-*VK4-116 and positive control, midazolam using human, rat, dog and monkey liver microsomes (% remaining Vs minutes; mean).

To determine the lysosomal perturbation potential of *R-*VK4-116, Lyso-ID Red Cytotoxicity kit, Enzo Sciences (Farmingdale, NY), Lot #03281735D was used with the manufacturer’s instructions. Briefly, the HepG2 cells seeded in 96-well plates were incubated with *R-*VK4-116 (0.1, 1.0, and 10.0 μM) for 24, 48 and 72 hr. Positive control verapamil (10, 100, and 200 μM) and negative control piroxicam were also included (Fig 2 in S1 File). After incubation timepoint, the treated cells were washed and incubated with fluorescent dual color detection reagent for 60 min. Subsequently, the fluorescence was measured with Spectramax ID5. An increase in red signal without significant loss (no more than 30%) of the blue signal indicates the accumulation of test article within the cells arising from an increase in lysosome or lysosome-like vesicles in size and/or number. The assay results were presented as fluorescence fold change for red stain and blue stain individually and were calculated as follows:

$$Fluorescence\;fold\;change=\frac{Relative\;Fluorescence\;Unit\;(RFU)\;measured\;for\;test\;article\;concentration}{RFU\;measured\;for\;respective\;solvent\;control}$$

The acceptance criteria for significant lysosomal perturbation was a ≥ 2-fold increase in red fluorescence with no loss of blue fluorescence.

### Mitochondrial toxicity (MT) assay

**Fig 3:**
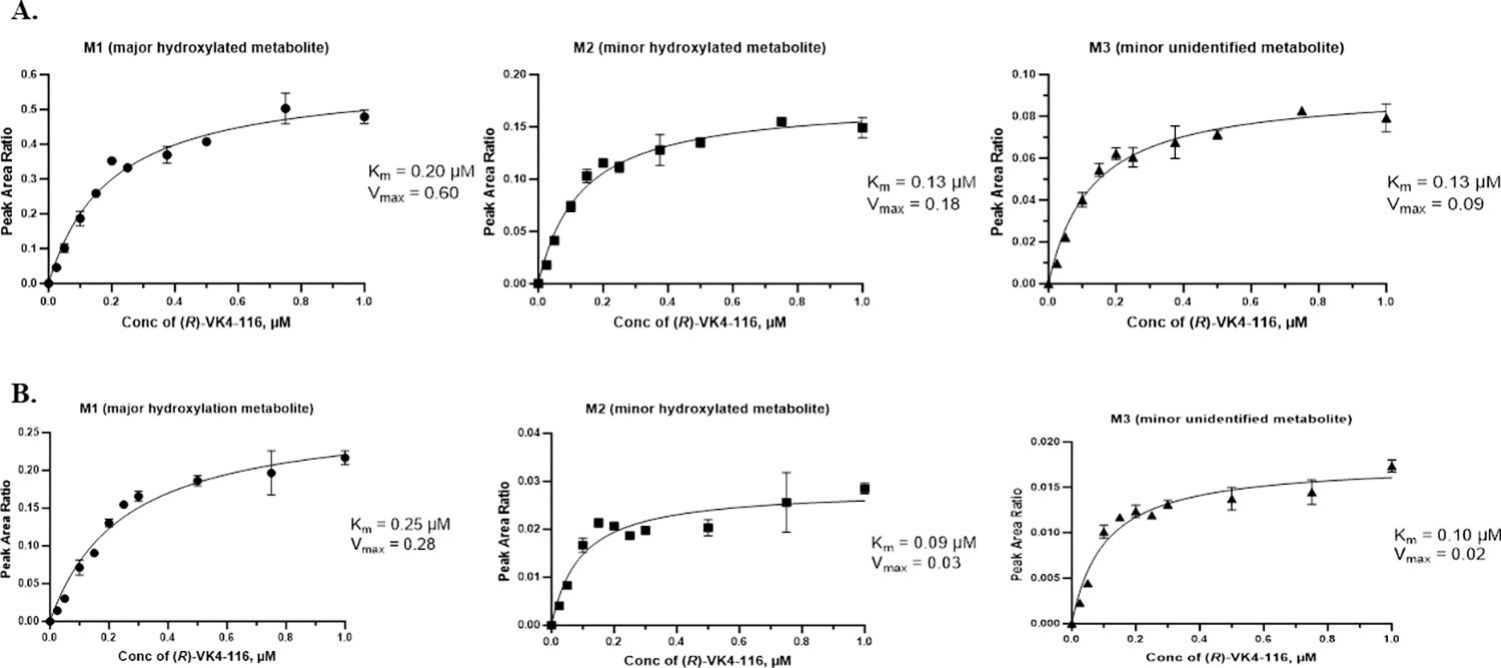
Kinetic plots for the formation of metabolites of VK4-116 by CYP2D6 (Fig 3A) and CYP3A4 (Fig 3B). M1, the major hydroxylated metabolite; M2, the minor hydroxylated metabolite; and M3, the minor unidentified metabolite.

Evaluation of the MT potential of *R-*VK4-116 was a two-step process. In the first step, the HepG2 cells were incubated with *R-*VK4-116 (0.001, 0.01, 0.1, 1, 10, 50 and 100 μM) for 24-, 48- and 72-hr to conduct cytotoxicity potential using MTT assay. The assay results were presented as % cell viability. In the second step, the MT potential was evaluated by treating HepG2 cells with *R-*VK4-116 (0.1, 1 and 10 μM) for 7- and 14-days of incubation. After each incubation, the cells were harvested and lysed to test the MT assay panel. The Zalcitabine (positive control) was also included in this assay (Fig 3 in S1 File). The levels of Mitochondrial Oxidative Phosphorylation (OXPHOS) complex 1 (NADH dehydrogenase; ab178011, lot #GR 3339973–1), complex 3 (CytC reductase; ab124537, lot #GR3276896-3), complex 4 (CytC Oxidase; ab179880, lot #GR3273194-1), and complex 5 (ATP synthase; ab124539, lot #GR3267780-4) in the cell lysates were determined using respective indirect ELISA as per manufacturer’s instructions [17]. The total ATP content of the cell lysates was determined using an ATP assay kit (ab83355, lot #GR3314128-2, Abcam, Cambridge, MA) [18]. The Caspase 3 content of the cell lysates was determined using the Caspase-3 assay kit (ab39401, lot #GR3322733-1, Abcam, Cambridge, MA) [19]. In addition, the total glutathione content was determined using the total glutathione (GSSG/GSH) assay kit (STA-312, lot #09100263, Oxiselect™, San Diego, CA) [20]. Using an Oxiselect™ ROS/RNS assay kit (STA-347, lot #7081343, Cell Biolabs Inc. San Diego, CA), the ROS/RNS content of the cell lysates was determined by fluorescence assay [21].

### Off-target enzyme effects, receptor functional assays and electrophysiological assays

**Fig 4:**
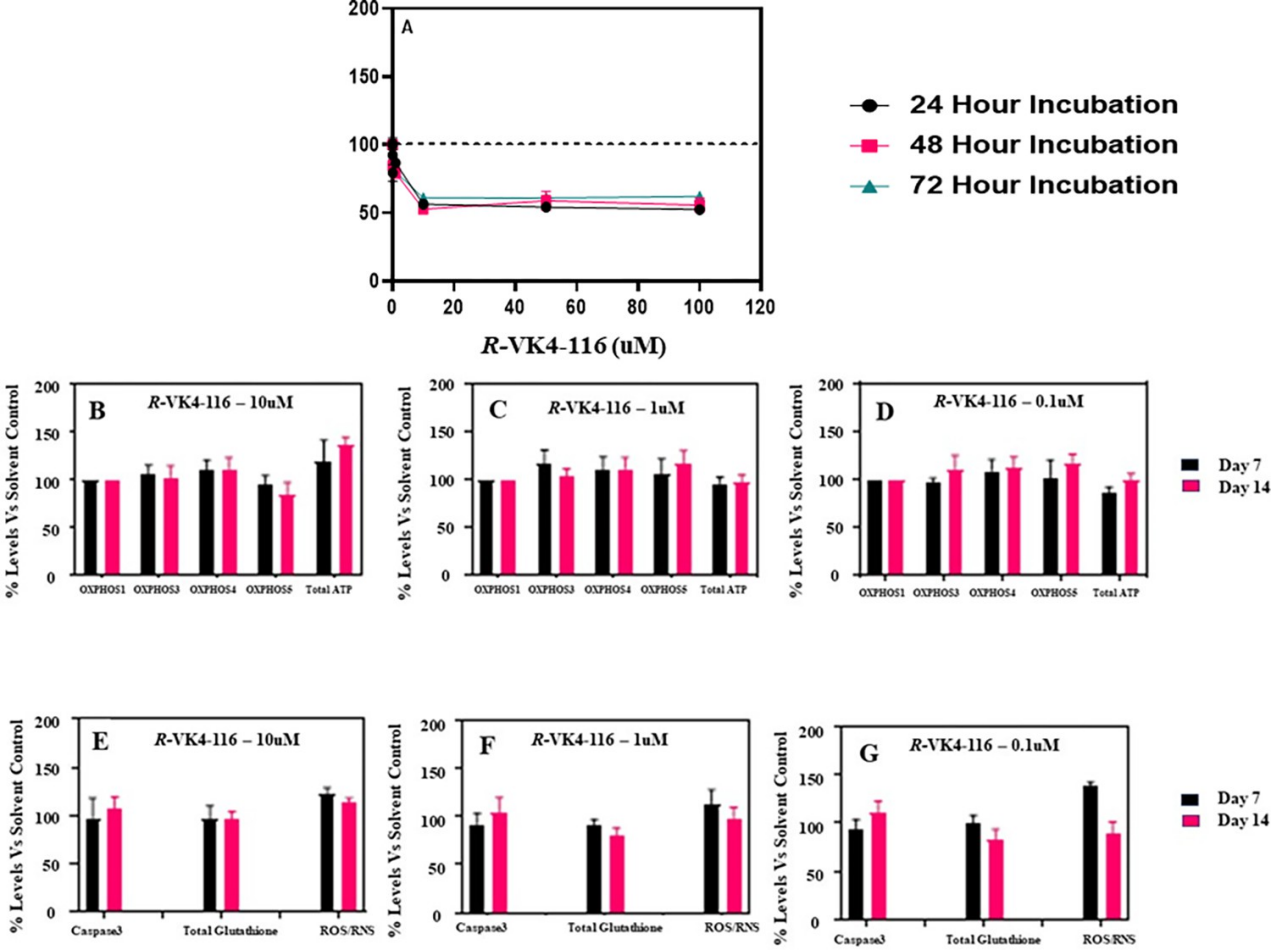
HepG2 cell viability (A), and MT with 10 μM (B, E), 1 μM (C, F) and 0.1 μM (D, G) of *R-*VK4-116.

The purpose of this study was to provide a first step in preliminary safety assessments by providing early identification of potential off-target interactions for the optimization of safety margins. This study was conducted by Eurofins Cerep (France) and Eurofins Discovery (St. Charles, MO). *R-*VK4-116 was tested in enzyme and uptake assay with 10 enzyme targets (Figs 4 and 5 in S1 File), and cellular and nuclear receptor functional assays with 24 receptor targets (Figs 5 and 6 in S1 File). Compound enzyme inhibition effect was calculated as a % inhibition of control enzyme activity. The cellular agonist effect was calculated as a % of the control response to a known reference agonist for each target, and the cellular antagonist effect was calculated as a % inhibition of the control reference agonist response for each target.

Electrophysiological assays were also conducted to profile *R-*VK4-116 for activities on ion channel targets. The QPatch HT (patch clamp) measured the agonist or antagonist activity of *R-*VK4-116 on ion channels. For these assays, the voltage was used as the ligand to activate the channel and induce a measurable current. The IonFlux HT measured the agonist, antagonist, or PAM activity of *R-*VK4-116 on ligand-gated ion channels. For these assays, GABA or Acetylcholine (ACh) was used as the ligand to activate the channel and induce measurable current. The six ion channels tested at 10 μM of *R-*VK4-116 in replicate were hNav1.5 Peak, hERG, hKCNQ1/minK, hCav1.2, hGABAA∞1/β2g2 and hnAChR∞4/β2.

### Comprehensive *in vitro* proarrhythmia assay (CiPA)

To evaluate proarrhythmic risk, *R-*VK4-116 (0.01, 0.1, 1, 10 and 100 μM) was tested in eight IonChannelProfiler™ Cardiac Panel Assays. Electrophysiological assays were conducted to profile *R-*VK4-116 for activities on the ion channel targets: Voltage-Gated Sodium: Nav1.5 (Peak), Voltage-Gated Potassium: hERG, KCNQ1/minK, Kv4.3/KChIP2, Kv1.5 Voltage-Gated Calcium: Cav1.2, Inward-Rectifying Voltage-Gated Potassium: Kir2.1 and Hyperpolarization-Activated Cyclic Nucleotide-Gated Potassium: HCN4 using the QPatch electrophysiological platform.

### KINOME^TM^ profiling

KINOME^TM^ profiling was done to quantitatively measure the interaction between *R-*VK4-116 and 403 non-mutant human kinases for screening off-target kinase activity. Briefly, *E*. *coli* host derived from the BL21 strain were grown to log-phase and infected with kinase-tagged T7 phage and incubated with shaking at 32°C until lysis. The lysates were centrifuged and filtered to remove cell debris. The remaining kinases were produced in HEK-293 cells and subsequently tagged with DNA for qPCR detection. Streptavidin-coated magnetic beads were treated with biotinylated small molecule ligands to generate affinity resins for kinase assays. The liganded beads were blocked with excess biotin and washed to remove unbound ligand and to reduce non-specific phage binding. To determine the small molecule (*R-*VK4-116) selectivity, the binding reactions were assembled by combining kinases, liganded affinity beads, and *R-*VK4-116 in 1x binding buffer [22]. *R-*VK4-116 was screened, and results for primary screen binding interactions were reported as ’% Ctrl’, where lower numbers indicated stronger hits in the matrix. Selectivity Score (S-score) was a quantitative measure of compound selectivity. It was calculated by dividing the number of kinases that compounds bind by the total number of distinct kinases tested, excluding mutant variants [22, 23]. The binding constants (Kds) were also determined with a standard dose-response curve using the Hill equation and Levenberg-Marquardt algorithm [24, 25].

### Plasma protein binding

The gel filtration used solid-phase extraction columns containing hydrated gel filtration beads (Sephadex G-25). *R-*VK4-116 (0.5 and 1.0 μM) was prepared in human, rat or dog plasma (final non-plasma matrix 1%), incubated at 37°C for 2 hr, and then determined the binding of *R-*VK4-116 to plasma protein by Sephadex G-25 gel filtration. In this method, the amount of analyte accompanying the proteins (i.e., protein-bound *R-*VK4-116) before and after filtration was assayed for analyte concentration by LC-MS/MS.

### Phospholipidosis and steatosis

To assess the potential of *R-*VK4-116 (≤100 μM) to cause phospholipidosis and steatosis in HepG2 cells, this study measured the effects of *R-*VK4-116 on the accumulation of phospholipids in the lysosomes of the cells and accumulation of fat in the cells, as well as mechanisms of toxicity as demonstrated by cell count, nuclear size, and DNA structure measurements using high content screening. Sertraline was included as a positive control for phospholipidosis, and tamoxifen was included as a positive control for steatosis.

### Statistical analysis

The data analysis and the figures were generated using GraphPad Prism Version 8.3 (GraphPad Software, San Diego CA). ViiA™7 v1.2.3 software (Applied Biosystems) was used for PCR data analysis. Analyst^®^ software v1.6.2 (Applied Biosystems) was used for LC-MS/MS data analysis. In the transporter mediated assay and MT assay, data were analyzed using Student’s t-test, where *p*-value <0.05 was statistically significant.

## Results

### Metabolic stability of *R-*VK4-116 in hepatocytes

Overall, *R-*VK4-116 (0.5 and 10 μM) was metabolically stable in human, rat, or dog hepatocytes. Although, some trend of metabolism was observed after 120 min of incubation at 0.5 μM, it was not substantial if compared to the stability of *R-*VK4-116 in the absence of hepatocytes after 120 min (Table 1; Fig 1). In the non-specific binding experiment, *R-*VK4-116 showed no significant loss after 5 min of incubation based on the pre-incubation conditions (Table A in S1 File).

**Table 1:** Metabolism of *R-*VK4-116 and positive control, midazolam using human, rat, and dog hepatocytes and in the absence of hepatocytes (% remaining Vs T = 0 min; mean).

Species^a^	Time (min.)	0.5 μM *R-*VK4-116	10 μM *R-*VK4-116	10 μM Midazolam
**Human**	0	100.0	100.0	100.0
	30	89.1	93.2	91.7
	60	78.4	85.5	85.3
	90	65.4	78.9	80.7
	120	48.1	79.7	72.4
**Rat**	0	100.0	100.0	100.0
	30	85.0	95.8	89.0
	60	55.6	92.5	77.8
	90	42.4	66.7	70.5
	120	42.5	64.6	65.7
**Dog**	0	100.0	100.0	100.0
	30	71.2	85.3	66.6
	60	61.2	85.5	50.8
	90	45.4	80.3	31.2
	120	40.1	76.4	19.5
**No hepatocytes** ^**b**^	0	100.0	100.0	100.0
	30	86.2	92.9	97.7
			
	60	81.6	87.5	97.6
	90	79.9	87.9	98.6
	120	78.4	77.3	98.5

^a^Suspension of hepatocytes of each species (human, rat and dog) was used in duplicate. The data is presented in mean.

^b^No hepatocytes were used. The results were presented in the mean of six sample results.

*In vitro* t_1/2_ and CL_int_ of *R-*VK4-116 are presented in Table 2. For *R-*VK4-116, human, rat, and dog hepatocytes showed similar t_1/2_ (~91 to 117 min at 0.5 μM and ~169 to 347 min at 10 μM), and CL_int_ (~12 to 16 μl/min/10^6^ cells at 0.5 μM and ~4 to 8 μl/min/10^6^ cells at 10 μM).

**Table 2:** *In vitro* half-life (t_1/2_) and intrinsic clearance (Cl_int_) of *R-*VK4-116 and positive control, midazolam using human, rat and dog hepatocytes.

Species	0.5 μM *R-*VK4-116		10 μM *R-*VK4-116		10 μM Midazolam	
	t_1/2,_ min	Cl_int_ μL/min/10^6^ cells	t_1/2,_ min	Cl_int_ μL/min/10^6^ cells	t_1/2,_ min	Cl_int_ μL/min/10^6^ cells
**Human**	117	11.8	338	4.1	267	5.2
**Rat**	86.6	16.0	169	8.2	193	7.2
**Dog**	91.2	15.2	347	4.0	51.3	27.0

### Metabolic stability and metabolite identification of *R-*VK4-116 in liver microsomes

The metabolism of *R-*VK4-116 was observed in human, rat, dog, or monkey liver microsomes after 30 min of exposure (Table 3; Fig 2). The extent of metabolism was as follows: 16.4%, 37.5%, 19.7%, and 15.0% remaining following 120 min exposure of 1 μM *R-*VK4-116 with human, rat, dog, and monkey microsomes, respectively; and 48.9%, 69.4%, 49.9%, and 39.7% remaining for 10 μM *R-*VK4-116, respectively. When incubated with HI microsomes, there was no significant decrease in *R-*VK4-116 after 120 min. Table 3 also presented the results for the positive control midazolam, which were consistent with expected values, demonstrating the integrity of the assay.

**Table 3:** Metabolic stability of *R-*VK4-116 and positive control, midazolam using human, rat, dog and monkey liver microsomes (% remaining Vs minutes; mean).

Species	Time (min.)	1 μM *R-*VK4-116	10 μM *R-*VK4-116	10 μM Midazolam	Species	Time (min.)	1 μM *R-*VK4-116	10 μM *R-*VK4-116	10 μM Midazolam
Human	0	100.0	100.0	100.0	Dog	0	100.0	100.0	100.0
	30	41.1	69.7	9.7		30	44.6	74.3	0.3
	60	25.9	60.1	1.0		60	22.5	64.5	0.1
	90	22.3	55.5	0.2		90	22.3	55.6	0.0
	120	16.4	48.9	0.0		120	19.7	49.9	0.0
*HI-Human	0	100.0	100.0	100.0	HI-Dog	0	100.0	100.0	100.0
	120	96.7	95.1	101.3		120	90.7	90.6	95.8
Rat	0	100.0	100.0	100.0	Monkey	0	100.0	100.0	100.0
	30	79.4	87.1	5.8		30	32.0	60.1	0.1
	60	56.8	79.7	0.4		60	21.2	46.5	0.0
	90	45.5	73.3	0.1		90	17.5	42.1	0.0
	120	37.5	69.4	0.0		120	15.0	39.7	0.0
HI-Rat	0	100.0	100.0	100.0	HI-Monkey	0	100.0	100.0	100.0
	120	94.9	95.4	102.2		120	90.0	89.9	91.8

*HI: Heat Inactivated

The relative PAR of the metabolites in the human, rat, dog, and monkey microsomes were shown in Table 4 to visualize the metabolites. The resulting metabolites were consistently detected in metabolic stability samples but not in controls. As seen in Table 4, four metabolites of *R-*VK4-116 have detected: two mono-oxidation products (M3 and M4), one di-oxidation product (M1), and one demethylated plus di-oxidation product (M2) metabolites. Similar metabolite profile (M2, M3 and M4) was seen in human and dog. Metabolite profile to some extent (M3) was also similar in human and rat. However, monkey showed different metabolite (M1).

**Table 4:** Metabolites of *R-*VK4-116 detected in 10 μM samples incubated with human, rat, dog, and monkey liver microsomes.

Species	ID	RT (min)	Mass Shift	Nominal Mass Change^a^ (m/z)	Precursor Form	Biotransformation	% Area
Human	M2	7.4	18	503.7 / 116.1	Demethylation + Di-Oxidation	[M-CH3+2O+H]*	4.0
	M3	7.4	16	501.7 / 116.1	Oxidation	[M+O+H]*	18.8
	M3^b^	7.4	16	501.7 / 144.1	Oxidation	[M+O+H]*	1.7
	M4	7.7	16	501.7 / 132.1	Oxidation	[M+O+H]*	5.6
	Parent	8.5	0	485.7 / 144.1	Parent	[M+H]*	4.1
	Parent	8.5	0	485.7 / 116.1	Parent	[M+H]*	62.4
Rat	M3	7.4	16	501.7 / 116.1	Oxidation	[M+O+H]*	5.8
	Parent	8.6	0	485.7 / 144.1	Parent	[M+H]*	86.1
	Parent	8.6	0	485.7 / 116.1	Parent	[M+H]*	8.2
Dog	M1	6.9	32	517.7 / 116.1	Di-Oxidation	[M+2O+H]*	4.1
	M2	7.4	18	503.7 / 116.1	Demethylation+ Di-Oxidation	[M-CH3+2O+H]*	44.5
	M3	7.4	16	501.7 / 116.1	Oxidation	[M+O+H]*	6.4
	M4	7.8	16	501.7 / 132.1	Oxidation	[M+O+H]*	6.1
	Parent	8.6	0	485.7 / 144.1	Parent	[M+H]*	28.6
	Parent	8.6	0	485.7 / 116.1	Parent	[M+H]*	10.4
Monkey	M1	6.9	32	517.7 / 116.1	Di-Oxidation	[M+2O+H]*	100

^a^Samples analyzed in Multiple Reaction Monitoring (MRM) mode; Precursor Ion → 116, 132, 144 Product Ions.

^b^Same retention time as M2 and M3, same precursor ion (501.7) but different product ion (144.1).

### CYP phenotyping

In the CYP phenotyping reaction, the greatest decrease of *R-*VK4-116 was observed when incubated with CYP2D6 and CYP3A4, with 40.6 and 25.6% remaining, respectively, at 120 min. Minimal to no metabolism was observed with the other CYP isoforms. A preliminary investigation of the metabolites formed resulted in the detection of three metabolites, two at a molecular mass of 500 (+16 amu, most likely hydroxylation of parent) and one at a molecular mass of 498 (+14 amu). Based on the PAR of metabolite to IS, one of the +16 metabolites appeared to be the major metabolite (M1), while the other two were minor metabolites (M2 and M3).

The K_m_ for the formation of M1, M2, and M3 by CYP2D6 was determined to be 0.20, 0.13, and 0.13 μM, respectively, while the V_max_ was 0.60, 0.18, and 0.09 (PAR of metabolite to IS), respectively (Table 5 and Fig 3). The K_m_ for the formation of M1, M2, and M3 by CYP3A4 was determined to be 0.25, 0.09, and 0.10 μM, respectively, while the V_max_ was 0.28, 0.03, and 0.02 (PAR of metabolite to IS), respectively (Table 5 and Fig 3).

**Table 5:** Metabolism of *R-*VK4-116 by CYP2D6 and CYP3A4– varying substrate concentrations for K_m_ and V_max_ determination.

*R-*VK4-116 (μM)	M1 (Major hydroxylated)		M2 (Minor hydroxylated)		M3 (Minor unidentified)	
	Peak Area Ratio		Peak Area Ratio		Peak Area Ratio	
	CYP2D6	CYP3A4	CYP2D6	CYP3A4	CYP2D6	CYP3A4
	Mean ± SD	Mean ± SD	Mean ± SD	Mean ± SD	Mean	Mean ± SD
0	0.000 **±** 0.000	0.000 **±** 0.000	0.000 **±** 0.000	0.000 **±** 0.000	0.000 **±** 0.000	0.000 **±** 0.000
0.025	0.0456 **±** 0.0075	0.0143 **±** 0.0005	0.0181 **±** 0.0019	0.00406 **±** 0.0002	0.00975 **±** 0.0011	0.00229 **±** 0.0001
0.05	0.102 **±** 0.0122	0.0303 **±** 0.0011	0.0414 **±**0.0018	0.00833 **±** 0.0001	0.0222 **±** 0.0014	0.00443 **±** 0.0002
0.1	0.187 **±** 0.0211	0.0714 **±** 0.0099	0.0740 **±** 0.0048	0.0167 **±** 0.0015	0.0401 **±** 0.0034	0.0101 **±** 0.0007
0.15	0.259 **±** 0.0072	0.0904 **±** 0.0007	0.103 **±** 0.0064	0.0214 **±** 0.0009	0.0544 **±** 0.0031	0.0117 **±** 0.0003
0.2	0.352 **±** 0.0029	0.130 **±** 0.0051	0.115 **±** 0.0029	0.0207 **±** 0.0001	0.0622 **±** 0.0028	0.0124 **±** 0.0007
0.25	0.333 **±** 0.0060	0.155 **±** 0.0047	0.111 **±** 0.0050	0.0187 **±** 0.0006	0.0605 **±** 0.0047	0.0119 **±** 0.0002
0.375	0.369 **±** 0.0234	0.166 **±** 0.0068	0.128 **±** 0.0147	0.0198 **±** 0.0008	0.0677 **±** 0.0078	0.0131 **±** 0.0005
0.5	0.407 **±** 0.0117	0.186 **±** 0.0067	0.135 **±** 0.0046	0.0203 **±** 0.0017	0.0714 **±** 0.0021	0.0137 **±** 0.0012
0.75	0.503 **±** 0.0447	0.197 **±** 0.0293	0.155 **±** 0.0026	0.0256 **±** 0.0062	0.0827 **±** 0.0015	0.0145 **±** 0.0014
1	0.479 **±** 0.0197	0.217 **±** 0.0091	0.149 **±** 0.0096	0.0285 **±** 0.0011	0.0793 **±** 0.0067	0.0173 **±** 0.0007

### CYP induction and inhibition

Due to the potential cytotoxicity of 30 μM *R-*VK4-116 in the initial MTT assay (Table B in S1 File), the highest concentration for the induction assay was reduced to 10 μM. For the concurrent MTT assay, no decrease of hepatocyte viability was measured in response to ≤ 10 μM *R-*VK4-116 in hepatocytes (Table C in S1 File).

Treatment of hepatocytes with *R-*VK4-116 (0.01 to 10 μM) for two days resulted neither in induction in CYP1A2 nor in CYP2B6 mRNAs and activities (Tables D-G in S1 File). However, treatment of hepatocytes with *R-*VK4-116 (0.01 to 10 μM) for two days resulted in a slight induction in CYP3A4 mRNA with an average 2.9-fold at 5 μM. However, this induction was not concentration-dependent throughout hepatocyte lots. The positive control showed significant induction (26-fold) in CYP3A4 mRNA. (Table 6). In addition, *R-*VK4-116 (0.01 to 10 μM) did not increase CYP3A4 activity (Table H in S1 File).

**Table 6:** Effect of *R-*VK4-116 on CYP3A4 mRNA expression in human hepatocytes.

Hepatocyte Lot No.	Treatment	Concentration (μM)	Fold Induction^a^
Lot 336	0.1% DMSO	0	1.0 ± 0.076
	*R-*VK4-116	0.01	1.1 ± 0.079
	*R-*VK4-116	0.1	1.1 ± 0.18
	*R-*VK4-116	1	2.3 ± 0.15
	*R-*VK4-116	3	3.7 ± 0.56
	*R-*VK4-116	5	4.0 ± 0.78
	*R-*VK4-116	10	3.7 ± 0.20
	0.1% DMSO	0	1.0 ± 0.19
	Rifampicin	10	37 ± 7.8
Lot 348B	0.1% DMSO	0	1.0 ± 0.17
	*R-*VK4-116	0.01	1.1 ± 0.050
	*R-*VK4-116	0.1	1.2 ± 0.081
	*R-*VK4-116	1	1.3 ± 0.12
	*R-*VK4-116	3	1.8 ± 0.17
	*R-*VK4-116	5	2.0 ± 0.27
	*R-*VK4-116	10	1.9 ± 0.13
	0.1% DMSO	0	1.0 ± 0.084
	Rifampicin	10	20 ± 3.0
Lot 399	0.1% DMSO	0	1.0 ± 0.025
	*R-*VK4-116	0.01	1.2 ± 0.20
	*R-*VK4-116	0.1	1.2 ± 0.11
	*R-*VK4-116	1	1.8 ± 0.12
	*R-*VK4-116	3	2.6 ± 0.14
	*R-*VK4-116	5	2.7 ± 0.40
	*R-*VK4-116	10	2.3 ± 0.39
	0.1% DMSO	0	1.0 ± 0.14
	Rifampicin	10	21 ± 3.6

Data are the mean ± SD from 3 wells.

^a^ Fold of vehicle control—the mean fold change of treated samples compared to vehicle control samples.

As per the Table I in S1 File, *R-*VK4-116 did not inhibit CYP significantly. Also, concentration dependent inhibition was not observed for any of the CYP450 isoforms screened when incubated with *R-*VK4-116 up to 100 μM. The positive inhibitor control yielded significant inhibition of CYP enzyme activities consistent with expected values, demonstrating the integrity of the assays.

### Transporter mediated assay

At 1 μM of *R-*VK4-116, less than a 2-fold difference in mean uptake rate was observed for each SLC transporter studied. Therefore, *R-*VK4-116 does not appear to be a substrate as defined by regulatory guidance documents [12] for human OAT1, OAT3, OCT2, OATP1B1, OATP1B3, MATE1, or MATE2-K under the study conditions (Fig 1 in S1 File). In addition, no statistically significant inhibition was seen for OAT1, OAT3, OCT2, OATP1B1, and OATP1B3 at 10 μM *R-*VK4-116. However, slight but significant inhibition (*p* <0.05) was seen for MATE1 (19.4% ± 7.57%), and MATE2-K (37.8% ± 9.26%) (Table 7).

**Table 7:** The inhibition of OAT1, OAT3, OCT2, OATP1B1, OATP1B3, MATE1 and MATE-2K mediated transport by *R-*VK4-116.

Transporter	Condition	Test Conditions	Accumulation of probe substrate (pmol/min/cm^2^)			Inhibition (%)	p value
			transporter cells	control cells	Net		
OAT1	No BSA	0.5% DMSO	1.64 ± 0.276	0.0855 ± 0.0113	1.56 ± 0.276		
		100 μM probenecid	0.198 ± 0.024	0.0790 ± 0.00983	0.119 ± 0.0240	92.4 ± 1.54	
	0.1% BSA	0.5% DMSO	2.09 ± 0.178	0.0741 ± 0.00739	2.02 ± 0.178		
		10 μM *R-*VK4-116	1.90 ± 0.0319	0.0645 ± 0.00739	1.84 ± 0.0319	8.97 ± 1.58	>0.05
OAT3	No BSA	0.5% DMSO	0.105 ± 0.00225	0.0116 ± 0.000391	0.0939 ± 0.00225		
		100 μM probenecid	0.0159 ± 0.00068	0.0116 ± 0.00130	0.00438± 0.00068	95.3 ± 0.73	
	0.1% BSA	0.5% DMSO	0.0483 ± 0.00484	0.00554 ± 0.000226	0.0428 ± 0.00484		
		10 μM *R-*VK4-116	0.0503 ± 0.00345	0.00500 ± 0.00150	0.0452 ± 0.00345	-5.73 ± 8.06	>0.05
OCT2	No BSA	0.5% DMSO	7.91 ± 0.660	0.576 ± 0.0332	7.34 ± 0.660		
		1000 μM quinidine	0.345 ± 0.0153	0.370 ± 0.0174	-0.0254 ± 0.0153	100 ± 0.21	
	0.1% BSA	0.5% DMSO	8.13 ± 0.627	0.531 ± 0.0436	7.6 ± 0.627		
		10 μM *R-*VK4-116	9.49 ± 0.143	0.566 ± 0.0544	8.93 ± 0.143	-17.4 ± 1.88	0.0234
OATP1B1	No BSA	0.5% DMSO	0.822 ± 0.101	0.0840 ± 0.00	0.738 ± 0.101		
		100 μM rifampicin	0.0687 ± 0.0101	0.0561 ± 0.0102	0.0126 ± 0.0101	98.3 ± 1.37	
	0.1% BSA	0.5% DMSO	0.672 ± 0.0148	0.0413 ± 0.00284	0.630 ± 0.0148		
		10 μM *R-*VK4-116	0.634 ± 0.109	0.0387 ± 0.00839	0.596 ± 0.109	5.50 ± 17.3	>0.05
OATP1B3	No BSA	0.5% DMSO	2.34 ± 0.136	0.0234 ± 0.00936	2.31 ± 0.136		
		100 μM rifampicin	0.0411 ± 0.00597	0.0234 ± 0.00519	0.0176 ± 0.00597	99.2 ± 0.26	
	0.1% BSA	0.5% DMSO	2.50 ± 0.181	0.00588 ± 0.00	2.49 ± 0.181		
		10 μM *R-*VK4-116	2.40 ± 0.266	0.0350 ± 0.0116	2.36 ± 0.266	5.29 ± 10.7	>0.05
MATE1	No BSA	0.5% DMSO	23.2 ± 1.07	0.463 ± 0.0174	22.7 ± 1.07		
		100 μM cimetidine	0.597 ± 0.0495	0.434 ± 0.0163	0.163 ± 0.0495	99.3 ± 0.22	
	0.1% BSA	0.5% DMSO	24.2 ± 1.25	0.431 ± 0.0725	23.7 ± 1.25		
		10 μM *R-*VK4-116	19.4 ± 1.80	0.290 ± 0.0259	19.1 ± 1.80	19.4 ± 7.57	0.0219
MATE2-K	No BSA	0.5% DMSO	5.48 ± 0.226	0.435 ± 0.0314	5.04 ± 0.226		
		100 μM cimetidine	0.738 ± 0.0296	0.531 ± 0.0866	0.208 ± 0.0296	95.9 ± 0.59	
	0.1% BSA	0.5% DMSO	5.93 ± 0.859	0.360 ± 0.0457	5.57 ± 0.859		
		10 μM *R-*VK4-116	3.76 ± 0.516	0.294 ± 0.0329	3.47 ± 0.516	37.8 ± 9.26	0.0221

In addition, results for the transcellular passage of *R-*VK4-116 were below the limit of quantitation in both the basolateral to apical (B->A) and in the apical to basolateral (A->B) directions for both ABC transporters (BCRP and P-gp) at 1 μM (Table J in S1 File). However, a significant inhibition was seen for BCRP (54.0% ± 1.46%) and P-gp (56.5% ± 6.17%) at 10 μM (Table 8). All probe substrates showed sufficient transport, and all reference inhibitors showed sufficient inhibition to meet the assay acceptance criteria.

**Table 8:** The inhibition of BCRP and P-gp mediated transport by *R-*VK4-116.

Transporter	Test Conditions	P_app_ B->A (x10^-6^ cm/s)	P_app_ A->B (x10^-6^ cm/s)	Mean Net B->A flux (pmol/min/cm^2^)	Efflux ratio (P_app_ B->A)/(P_app_ A->B)	Inhibition (%)	P value
BCRP	0.5% DMSO	53.4 ± 2.06	3.91 ± NA	6.95 ± 0.29	13.7 ± 0.53	-	-
	10 μM *R-*VK4-116	50.5 ± 1.36	7.40 ± 2.12	6.05 ± 0.19	6.82 ± 0.18	54.0 ± 1.46	<0.0001
	1 μM Ko143	21.2 ± 0.51	17.8 ± 0.83	0.48 ± 0.07	1.19 ± 0.03	98.5 ± 0.22	-
P-gp	0.5% DMSO	72.2 ± 16.2	1.10 ± 0.22	0.44 ± 0.1	65.6 ± 14.7	-	-
	10 μM *R-*VK4-116	74.7 ± 10.2	2.57 ± 0.62	0.44 ± 0.06	29.1 ± 3.99	56.5 ± 6.17	0.0144
	3 μM elacridar	19.9 ± 3.38	9.87 ± 1.03	0.06 ± 0.02	2.01 ± 0.34	98.4 ± 0.53	-

The probe substrates were 2 μM prazosin for BCRP, and 0.1 μM quinidine for P-gp.

NA: One of the triplicates was removed by Grubbs test; standard deviation is not reported for this value.

Data represented the mean and standard deviation of triplicate samples.

### Lysosomal perturbation

The results from the lysosomal trapping assay were demonstrated in Fig 2 in S1 File. No significant increase in red lysosomal stain signal or decrease in blue nuclear stain signal over respective solvent control was observed for *R-*VK4-116 (Fig 2C in S1 File). The assay negative control piroxicam result was as expected (Fig 2B in S1 File). In contrast, a 2-3-fold increase in red lysosomal signal with no significant decrease of the blue nuclear signal was observed in HepG2 cells following 72-hr treatment with the two highest concentrations of the positive control verapamil (Fig 2A in S1 File). These results suggested that there was no significant lysosomal perturbation in HepG2 cells following 72-hr incubations of *R-*VK4-116 (0.1, 1.0, and 10.0 μM) over control.

### MT assay

The results from the cytotoxicity (MTT assay) are summarized in Fig 4A. Approximately 40–50% decrease in HepG2 cell viability compared with the solvent control was observed following 72-hr treatment with the *R-*VK4-116 (10 to 100 μM).

Based on the cell viability data, the three concentrations 0.1, 1 and 10μM of *R-*VK4-116 were selected for the subsequent 2-week MT assay. Fig 4B-4G summarized the results of the MT assay panel following 7- and 14-day treatment of HepG2 cells with *R-*VK4-116. No significant decrease in OXPHOS complex 1, 3, 4, and 5 or in total ATP levels was observed for any tested concentrations of *R-*VK4-116 (Fig 4B-4D). Furthermore, no significant increase in caspase 3, or total glutathione levels was observed for any tested concentrations of *R-*VK4-116. However, a 10–35% increase in ROS/RNS levels compared with the solvent control was observed for HepG2 cells treated for 7-days with all three concentrations of *R-*VK4-116 (Fig 4E-4G). The positive control, zalcitabine results are briefly described in Fig 3 in the S1 File.

### Off-target enzyme effects, receptor functional assays, electrophysiological assays and CiPA

An inhibition or stimulation higher than 50% was considered a significant effect, while results showing inhibition or stimulation between 25 and 50% were indicative of weak to moderate effects of the test compound. *R-*VK4-116 (10 μM) did not show any enzyme inhibition effects or uptake (Fig 4 in S1 File). In addition, *R-*VK4-116 did not exhibit agonist and antagonist effects for most of the targets (Figs 5 and 6 in S1 File). However, a significant inhibition (antagonist) effect of *R-*VK4-116 (10 μM) was observed for α1A*(h)* at 82.9% and H1*(h)* at 86.1%, while a weak to moderate antagonist effects were observed for 5-HT2B*(h)* at 44.6% and V1a*(h)* at 27.8%. Furthermore, *R-*VK4-116 showed an agonist effect of 97.71% for hGABAA∞1β2γ2 at 10 μM in the IonFlux HT.

In the CiPA assay, *R-*VK4-116 did not show any effect on the ion channels except hERG and Kri2.1. The hERG at 10 μM (54.60%) and 100 μM (82.26%), and Kir2.1 at 100 μM (Peak, 52.64% and End, 97.19%) showed an inhibition greater than 50% (Table K in S1 File).

### KINOME^TM^ profiling

S-score with %Control <35 results showed for the genes: Checkpoint kinase 2 (CHEK2), Hematopoietic progenitor kinase 1 (HPK1), Microtubule affinity-regulating kinase 3 (MARK3), Serine/threonine-protein kinase SRPK2 (SRPK2), and Tyrosine Kinase Non-Receptor 1 (TNK1). A follow-up KINOMEscan™ was performed with *R-*VK4-116 at an 11-point concentration-response curve ranging from 0.001 to 10 μM. These concentrations were tested at 5 hits that had a selectivity score of S (35) in the primary screen. *R-*VK4-116 interacted with CHEK2, HPK1, MARK3, SRPK2 and TNK1 (5 hits) at 1 μM. In all 5 hits, the binding constants (Kds) were >10 μM (Table L in S1 File).

### Plasma protein binding assay

Table 9 shows the results of the gel filtration protein binding experiment of *R-*VK4-116 in plasma. In human plasma, the mean percentage of *R-*VK4-116 bound to proteins was 97.2 and 85.9%, at 0.5 and 1 μM, respectively. In rat plasma, 83.2 and 92.8% of *R-*VK4-116 were bound to proteins when tested at 0.5 and 1 μM, respectively. Further, 83.6 and 82.7% of *R-*VK4-116 was bound to dog plasma proteins when tested at 0.5 and 1 μM, respectively.

**Table 9:** Determination of 0.5 and 1 μM *R-*VK4-116 binding to human, rat and dog plasma proteins by gel filtration.

Species	Nominal Conc. (μM)^a^	[*R-*VK4-116] Before Filtration (μM)	[*R-*VK4-116] In Sephadex G-25 Eluate (μM)	Ratio [*R-*VK4-116] Before Filtration / Eluate	Mean ± SD	% Bound^c^	Mean ± SD
**Human**	**0.5**	0.550	0.520	1.06	0.988 ± 0.13	97.4	97.2± NC
		0.597	0.715	0.835^b^		NCb	
		0.598	0.557	1.07		96.9	
**Rat**	**0.5**	0.631	0.533	1.18	1.19 ± 0.01	83.6	83.2±3.72
		0.631	0.535	1.18		79.3	
		0.630	0.525	1.20		86.7	
**Dog**	**0.5**	0.609	0.491	1.24	1.25 ± 0.01	77.9	83.6±7.50
		0.618	0.495	1.25		92.1	
		0.616	0.493	1.25		80.8	
**Human**	**1**	0.923	0.614	1.50	1.56 ± 0.06	92.5	85.9± NC
		0.995	0.615	1.62		81.6	
		1.02	0.657	1.55		83.7	
**Rat**	**1**	1.03	0.632	1.63	1.60 ± 0.02	91.4^d^	92.8±1.29
		1.01	0.632	1.60		93.9	
		1.02	0.647	1.58		93.2	
**Dog**	**1**	1.05	0.626	1.68	1.65 ± 0.13	73.3	82.7±9.60
		1.07	0.707	1.51		92.5	
		1.07	0.604	1.77		82.4	

^a^ Lower Limit of Quantitation (LLOQ) was 0.01 μM

^b^ Grubb’s test rejects outlier data, Not calculated

^c^ $\%Bound=\left(\frac{{\left[Protein\right]}_{Before}}{{\left[Protein\right]}_{Elute}}\right)\times \left(\frac{{\left[Drug\right]}_{Elute}}{{\left[Drug\right]}_{Before}}\right)\times 100$

^d^ % bound calculated using mean protein ratio since individual value could not be calculated

### Phospholipidosis and steatosis

Based on the results (Table 10), *R-*VK4-116 caused phospholipidosis (MEC of 0.0317 μM; AC50 of 10.7 μM). A steatosis response was also reported (MEC of 14.2 μM; AC50 of >100 μM); however, it was not considered significant since the response was just slightly above the significant cut-off from the vehicle control, and no dose response was observed for steatosis with increasing concentrations. No response was observed for cell count, nuclear size or DNA structure. For positive control sertraline, the lowest MEC and AC_50_ response indicated an increase in phospholipidosis. In the case of positive control tamoxifen, the lowest MEC and AC_50_ response indicated an increase in steatosis.

**Table 10:** Cell health parameters for *R-*VK4-116, sertraline and tamoxifen.

Parameter	*R-*VK4-116			Sertraline			Tamoxifen		
	Trend (*↑↓*)	MEC (μM)	AC_50_ (μM)	Trend (*↑↓*)	MEC (μM)	AC_50_ (μM)	Trend (*↑↓*)	MEC (μM)	AC_50_ (μM)
**Cell count**		NR	NR	*↓*	1.61	2.48	*↑*	0.360	0.442
**Nuclear size**		NR	NR		NR	NR	*↓*	0.672	3.05
**DNA structure**		NR	NR	*↑*	0.876	6.23	*↑*	2.57	6.30
**Phospholipidosis**	*↑*	0.0317	10.7	*↑*	*<*0.0977	0.351	*↑*	0.447	0.764
**Steatosis**	*↑*	14.2 (NS)	*>*100# (NS)	*↑*	0.822 (NS)	*>*3.13# (NS)	*↑*	4.38	16.9

MEC: Minimum effective concentration that significantly crosses vehicle control threshold.

AC_50_: The concentration at which 50% maximum effect is observed for each cell health parameter.

#An AC_50_ was calculated but is greater than the maximum surviving concentration.

NS: Not statistically significant.

**↑** increasing trend of response; **↓** decreasing trend of response

NR: No response observed.

## Discussion

D_3_R partial agonists and antagonists are being developed for the treatment of SUD (substance use disorder), including OUD. In one study, an acute dose (0.5 mg) of a D3 agonist did not reduce motivation for smoking cigarettes, while a 3 mg dose intensified cocaine effects in humans [26, 27]. Thus, the use of a D_3_R agonist for the treatment of nicotine dependence and cocaine use disorder is questionable. Acute and chronic treatments with the D_3_R antagonist (PG01037) but not the agonist (PF-592,379) in rats showed decreases in cocaine induced behaviors [28]. However, clinical translation of D3 antagonists has been challenging due to lack of favorable ADMET (Absorption, Distribution, Metabolism, Excretion and Toxicity) properties [3, 4]. VK4-116 (a new highly selective D_3_R antagonist) was designed to improve upon these challenges [5]. In addition, the enantioselective synthesis of *R*- and *S*-VK4-116 was achieved, and the *R*-enantiomer was determined to be the eutomer at D_3_R [8]. *R-*VK4-116 was effective in decreasing oxycodone self-administration, suppressing reinstatement to oxycodone-stimulated drug seeking, and, reducing withdrawal-associated hyperalgesia [6, 7]. In the past, the D_3_R-selective antagonist, GSK598,809 showed promising preclinical data for treatment of nicotine dependence and cocaine use disorder but further development of this agent was halted due to the development of hypertension in dogs in combination with cocaine [4, 9]. In addition, another D_3_R antagonist (SB-277,011A) also showed adverse cardiovascular effects in the presence of cocaine in dogs [29]. However, *R*-VK4-116 did not increase the cardiovascular effects of either cocaine or oxycodone in rats [9].

Methadone, buprenorphine, and naltrexone are all FDA-approved drugs for the treatment of OUD. However, novel and safe therapeutics would be a welcome addition to the medication toolbox, especially effective medications that are non-opioid. In this exploratory study, the highly D_3_R-selective antagonist, *R-*VK4-116, was evaluated *in vitro* to predict its safety *in vivo*, and to assess the relative sensitivity of different species to this compound. These studies were also important to choosing an appropriate animal species in which to conduct *in vivo* toxicology experiments (https://www.fda.gov/media/72028/download).

Drug metabolism mainly occurs in the liver [12]. So, metabolic stability was evaluated in the *in vitro* hepatocytes or liver microsomes. It is crucial to determine whether significant species differences are observed in drug metabolism [30]. This is also helpful to select animal species (one rodent and one non-rodent) with similar physiology that can produce similar metabolic stability and metabolites to humans for assigning the ‘relevant animal species’ for non-clinical safety evaluations [31, 32]. Rats and dogs are commonly used species for standard toxicity studies [32]. We evaluated *in vitro* metabolism of *R-*VK4-116 in human, rat and dog hepatocytes to predict *in vivo* metabolic intrinsic clearance [33]. *R-*VK4-116 was metabolically stable in the hepatocytes of each tested species (Table 1). The observed *in vitro* t_1/2_ and CL_int_ in rat, human and dog hepatocytes showed similar stability of *R-*VK4-116 (Table 2). In the metabolic stability study of *R-*VK4-116 using liver microsomes, the metabolism was similar in human, monkey, rat and dog (Table 3). Also, metabolites of *R-*VK4-116 observed in human were present in dog (M2, M3 and M4) and rat (M3) but not in monkey (M1). It is important to assess human-relevant metabolites adequately. Since rat and dog were showing similar metabolic stability and metabolite profile to human these animals can be used as appropriate species, while monkey may not be the appropriate species to conduct safety evaluations due to the difference in metabolite profile.

Since patients being treated with *R-*VK4-116 are likely to be prescribed other medications, it is essential to assess whether *R-*VK4-116 is a substrate, inducer or inhibitor in metabolism/transporter-mediated drug interactions. Several CYP enzymes involved in drug metabolism contribute to variable drug exposure levels [34, 35]. As per USFDA, CYP1A2, CYP2B6, CYP2C8, CYP2C9, CYP2C19, CYP2D6, and CYP3A4 should be used for *in vitro* phenotyping to determine enzymes involved in the metabolism of the investigational drug. If the investigational drug did not undergo significant metabolism by these major enzymes, additional enzymes can be evaluated for *in vitro* phenotyping [12]. In the phenotyping screen, CYP2D6 or CYP3A4 showed the greatest decrease in *R-*VK4-116 over time, which indicated a major contribution of these enzymes in metabolism (Table 5). In addition, CYPs induction and inhibition were major causes of clinical drug–drug interactions [36]. As per USFDA, *R-*VK4-116 was evaluated to assess its potential to induce (CYP1A2, CYP2B6 and CYP3A4) and inhibit (CYP1A2, CYP2B6, CYP2C8, CYP2C9, CYP2C19, CYP2D6, and CYP3A) enzymes [12]. *R-*VK4-116 caused a slight induction (2.9-fold) in CYP3A4 mRNA at a higher concentration (5 μM) as compared with the positive control, Rifampicin (26-fold induction). Also, this induction was neither concentration-dependent nor consistent in different lots of hepatocytes (Table 6). Further, *R-*VK4-116 did not cause induction in any CYP activity. Considering these results, *R-*VK4-116 was not evaluated further to assess its induction effect on other enzymes. In addition, the concentration dependent inhibition of *R-*VK4-116 was not observed for any of the CYP isoforms (Table I in S1 File). In clinical use, OAT1, OAT3, OCT2, OATP1B1, OATP1B3, MATE1, MATE-2K, P-gp and BCRP interact with drugs [12]. All uptake transporters (OAT1, OAT3, OCT2, OATP1B1 and OATP1B3) and two export transporters (MATE1 and MATE-2K) belong to the SLC transporters [37]. Most efflux transporters, including P-gp and BCRP, are part of the ABC transporter family [38]. Since these transporters can regulate the drug’s disposition and pharmacological action, the evaluation of *R-*VK4-116 is important as a substrate and/or inhibitor of these transporters [12]. Based on the result, *R-*VK4-116 was not a substrate for any transporters (Fig 1 and Table J in S1 File). Although, significant inhibition (>50%) was seen for BCRP and P-gp (Table 8), the concentration of *R-*VK4-116 was 10 μM, which is too high to be clinically relevant to cause the transporter-mediated drug interactions. P-gp or BCRP inhibition elevates the P-gp or BCRP substrate drug’s plasma concentration, respectively, which could lead to increased toxicity of the substrate [39–41]. Collectively, these results suggested that *R-*VK4-116 has a very low potential to cause metabolism/transporter-mediated drug interactions.

The protein binding ability may impact drug toxicity. Interspecies differences in plasma protein binding may lead to decreased or increased drug-safety margin [42]. In this assay, *R-*VK4-116 was bound to plasma in all tested species (~83–93%) (Table 9). This limits the partitioning of drug from blood to tissue, which reduces the burden of tissue/organ-specific toxicities. A drug’s cytotoxic effects are often first seen as lysosome perturbation, which is a result of ion trapping of amine-containing compounds to form autophagosomes and autophagic cytopathology [43]. The result in Fig 2C in S1 File suggested that *R-*VK4-116 did not show any cytotoxic effects. Since phospholipidosis and steatosis were considered adverse findings, it is important to identify lipid metabolic disorders induced by drugs in the early development stage [44] with *in vitro* cell-based assays: liver phospholipidosis and steatosis. The results demonstrated that *R-*VK4-116 did not induce significant phospholipidosis or steatosis (≤10 μM) (Table 10). Further, mitochondria are important in the production of cellular energy, fatty acid metabolism, calcium signaling, steroid synthesis, heme production, apoptosis and autophagy [45, 46]. Hence, drug-instigated mitochondrial dysfunction is implicated in organ toxicity. This can lead to drug withdrawal or black box warnings [47]. As per OXPHOS enzyme assays (energy metabolism disruption), ROS/RNS production (free radical production), total glutathione expression and caspase 3 activation (altered apoptosis) results (Fig 4B-4G), *R-*VK4-116 did not induce mitochondrial toxicity. Collectively, these results demonstrate the promising *in vitro* safety profile of *R-*VK4-116.

In human physiology, kinases are important for phosphorylation [48]. Kinases have major significance in metabolism, cell signaling, protein regulation, cellular transport and secretory processes [49, 50]. *R-*VK4-116 showed only 5 hits (CHEK2, HPK1, MARK3, SRPK2 and TNK1). CHEK2 plays role in the pathway of DNA damage repair [51] while HPK1 plays a role in stress responses, apoptosis and proliferation in hematopoietic cells [52]. MARK3 regulates cell cycle and cell differentiation [53]. SRPK2 contributes interaction and localization of pre-mRNA splicing factors in cells [54], while TNK1 plays a role in lymphoid cell proliferation [55]. However, the binding constants (Kds) for these 5 hits were >10 μM of *R-*VK4-116, which suggests the low potential to cause off-target kinase activity. Also, multiple assays were being used to examine the modulation of cellular and nuclear receptor functions [56]. In these assays, *R-*VK4-116 (10 μM) did not show any effects on the tested targets except the antagonist effect on α1A*(h)* and H1*(h)*, and the agonist effect on hGABAA∞1β2γ2. α1A*(h)* inhibition leads to a reduction in smooth muscle contraction [57], while H1(*h*) inhibition can cause somnolence or adverse effects on CNS [58]. Also, hGABAA activation may mediate sedative effects [59]. Since *R-*VK4-116 concentration was 10 μM in these assays, this concentration is too high to be clinically relevant to cause the off-target effects. CiPA helps to understand torsadogenic mechanisms beyond hERG blockade to evaluate proarrhythmic risk [60]. *R-*VK4-116 did not affect ion channels except hERG and Kri2.1. hERG controlled the QT interval, while Kir2.1 regulated the resting membrane potential and modulated excitability [61, 62]. Although *R-*VK4-116 inhibited the hERG channel, the inhibition was moderate to high (~54–82% at ≥10 μM). Also, *R-*VK4-116 inhibited the Kir2.1 channel at 100 μM. The concentration of *R-*VK4-116 was too high to be clinically relevant in the above assays. In addition, this compound did not potentiate the cardiovascular effects alone and in combination with either cocaine or oxycodone in rats [9]. Overall, these *in vitro* results indicated that *R-*VK4-116 has low potential to cause any impact on off-target kinase activity, off-target effects or changes in ion channel activities.

*R*-VK4-116 showed an antagonist effect on D_3_R at 350 nM (IC_50_), while the binding affinity for D_3_R was 7.4 nM (Ki) [8]. Further, 10 mg/kg oral dose of *R*-VK4-116 in rats showed C_max_ 454.4 pM/mL [8]. Considering these concentrations, the *in vitro* concentration (≥ 10 μM) that showed off-target effects in some assays will not be physiologically relevant. Although, these *in vitro* data are promising, this study has limitation of *in vivo* ADMET data. These *in vitro* results may not necessarily correlate with an *in vivo* result due to the absence of identical conditions for live organism’s cell [63]. However, this *in vitro* study results will help to design the *in vivo* studies with appropriate endpoints. For further development, rat (rodent) and dog (non-rodent) species are selected for planned GLP *in vivo* safety studies as a part of investigational new drug application (IND) submission to support human clinical trials.

In conclusion, the results of these *in vitro* studies indicated that *R-*VK4-116 possesses favorable safety properties with low potential to cause off-target toxicity to support its further development as a medication for OUD.

## Supporting information

S1 File Additional tables and figures. (DOCX)
