# Supplementary material for: In vitro safety evaluation of dopamine D3R antagonist, R-VK4-116, as a potential medication for the treatment of opioid use disorder
Source: PLoS One. 2024 Dec 16;19(12):e0315569. doi: 10.1371/journal.pone.0315569 (PMC11649115; doi:10.1371/journal.pone.0315569)
Supplement: S1 File — (DOCX) [file pone.0315569.s001.docx]

**Supporting Information**

**Non-Specific Binding Assessment**

*R-*VK4-116 was tested for non-specific binding to the assay were at 1 and 10 µM. Solutions were prepared in glass and three aliquots of each solution were taken for LC/MS/MS analysis (“Pre-Incubation Solutions”). Aliquots of these solutions were also placed into three wells of a 96-well culture plate without cells and incubated at 37^o^C for 5 minutes. Samples of each were taken following the incubations, and *R-*VK4-116 was quantified by LC/MS/MS (“Post-Incubation Solutions”).

**Table A. Results of Non-Specific Binding Assessment of *R-*VK4-116 (Data were represented by the mean and standard deviation of triplicate samples)**

| ***R-*VK4-116** | **Pre-Incubation Solutions** | **Post-Incubation Solutions** | **Pre-Incubation Solutions** | **Post-Incubation Solutions** |
| --- | --- | --- | --- | --- |
| **(µM)** | **(µM)** | **(µM)** | **(% of Nominal)** | **(% of Nominal)** |
| 1 | 0.987 ± 0.0358 | 0.973 ± 0.0288 | 98.7 ± 3.58 | 97.3 ± 2.88 |
| 10 | 7.02 ± 0.202 | 7.20 ± 0.0777 | 70.2 ± 2.02 | 72.0 ± 0.777 |

Results indicate reasonable recovery of *R-*VK4-116.

**CYP Induction Assay**

#### Table B. Effect of *R-*VK4-116 on Hepatocyte Viability as Measured by the MTT Assay Upfront

| **Treatment** | **Conc.**  **[µM]** | **Hepatocyte**  **(Mean** ± **SD)** | | |
| --- | --- | --- | --- | --- |
| 0.1% DMSO | 0 | 100 | ± | 14 |
| *R-*VK4-116 | 0.01 | 93 | ± | 18 |
| *R-*VK4-116 | 0.1 | 105 | ± | 15 |
| *R-*VK4-116 | 1 | 86 | ± | 13 |
| *R-*VK4-116 | 3 | 91 | ± | 14 |
| *R-*VK4-116 | 10 | 73 | ± | 11 |
| *R-*VK4-116 | 30 | 73 | ± | 9 |
| Tamoxifen | 50 | 4 | ± | 1 |

#### Table C. Effect of *R-*VK4-116 on Hepatocyte Viability as Measured by the Concurrent MTT Assay

| **Treatment** | **Conc. [µM]** | **Hepatocyte Lot 336**  **(Mean** ± **SD)** | | | **Hepatocyte Lot 348B**  **(Mean** ± **SD)** | | | **Hepatocyte Lot 399**  **(Mean** ± **SD)** | | |
| --- | --- | --- | --- | --- | --- | --- | --- | --- | --- | --- |
| 0.1% DMSO | 0 | 100 | ± | 12 | 100 | ± | 8 | 100 | ± | 6 |
| *R-*VK4-116 | 0.01 | 96 | ± | 14 | 100 | ± | 4 | 107 | ± | 3 |
| *R-*VK4-116 | 0.10 | 90 | ± | 7 | 101 | ± | 0 | 104 | ± | 5 |
| *R-*VK4-116 | 1 | 91 | ± | 7 | 104 | ± | 4 | 95 | ± | 4 |
| *R-*VK4-116 | 3 | 80 | ± | 4 | 96 | ± | 6 | 88 | ± | 3 |
| *R-*VK4-116 | 5 | 75 | ± | 4 | 97 | ± | 6 | 86 | ± | 6 |
| *R-*VK4-116 | 10 | 73 | ± | 4 | 102 | ± | 5 | 91 | ± | 6 |
| Tamoxifen | 50 | 2 | ± | 1 | 1 | ± | 1 | 7 | ± | 8 |

Table D. Effect of *R-*VK4-116 on CYP1A2 mRNA expression in human hepatocytes.

| **Hepatocyte**  **Lot No.** | **Treatment** | **Concentration** | **Fold Inductiona** | | |
| --- | --- | --- | --- | --- | --- |
|  |  | **[µM]** |  |  |  |
| Lot 336 | 0.1% DMSO | 0 | 1.0 | ± | 0.17 |
|  | *R-*VK4-116 | 0.01 | 1.1 | ± | 0.073 |
|  | *R-*VK4-116 | 0.1 | 1.2 | ± | 0.11 |
|  | *R-*VK4-116 | 1 | 1.6 | ± | 0.15 |
|  | *R-*VK4-116 | 3 | 1.9 | ± | 0.076 |
|  | *R-*VK4-116 | 5 | 1.8 | ± | 0.12 |
|  | *R-*VK4-116 | 10 | 1.9 | ± | 0.17 |
|  | 0.1% DMSO | 0 | 1.0 | ± | 0.17 |
|  | Omeprazole | 50 | 115 | ± | 8.2 |
| Lot 348B | 0.1% DMSO | 0 | 1.0 | ± | 0.10 |
|  | *R-*VK4-116 | 0.01 | 1.1 | ± | 0.18 |
|  | *R-*VK4-116 | 0.1 | 1.4 | ± | 0.089 |
|  | *R-*VK4-116 | 1 | 1.3 | ± | 0.20 |
|  | *R-*VK4-116 | 3 | 1.5 | ± | 0.15 |
|  | *R-*VK4-116 | 5 | 1.6 | ± | 0.24 |
|  | *R-*VK4-116 | 10 | 1.5 | ± | 0.10 |
|  | 0.1% DMSO | 0 | 1.0 | ± | 0.10 |
|  | Omeprazole | 50 | 361 | ± | 22 |
| Lot 399 | 0.1% DMSO | 0 | 1.0 | ± | 0.043 |
|  | *R-*VK4-116 | 0.01 | 1.6 | ± | 0.17 |
|  | *R-*VK4-116 | 0.1 | 1.8 | ± | 0.11 |
|  | *R-*VK4-116 | 1 | 1.7 | ± | 0.11 |
|  | *R-*VK4-116 | 3 | 1.9 | ± | 0.052 |
|  | *R-*VK4-116 | 5 | 1.6 | ± | 0.34 |
|  | *R-*VK4-116 | 10 | 1.6 | ± | 0.26 |
|  | 0.1% DMSO | 0 | 1.0 | ± | 0.043 |
|  | Omeprazole | 50 | 232 | ± | 37 |

Data are the mean ± SD from 3 wells

^a^ Fold of vehicle control - the mean fold change of treated samples compared to vehicle control samples.

**Table E. Effect of *R-*VK4-116 on CYP1A2 induction activity in human hepatocytes**

| **Hepatocyte Lot No.** | **Treatment** | **[µM]** | **Phenacetin-O- deethylation** | | | **Fold of Vehicle Controla** | | |
| --- | --- | --- | --- | --- | --- | --- | --- | --- |
|  |  |  | **pmol/min/106 cells** | | |  |  |  |
| Lot 336 | 0.1% DMSO | 0* | 1.2 | ± | - |  | - |  |
|  | *R-*VK4-116 | 0.01 | 1.1 | ± | 0.25 | 0.96 | ± | 0.22 |
|  | *R-*VK4-116 | 0.1 | 1.1 | ± | 0.19 | 0.96 | ± | 0.16 |
|  | *R-*VK4-116 | 1 | 1.4 | ± | 0.22 | 1.2 | ± | 0.19 |
|  | *R-*VK4-116 | 3 | 1.8 | ± | 0.35 | 1.5 | ± | 0.30 |
|  | *R-*VK4-116 | 5 | 1.8 | ± | 0.55 | 1.6 | ± | 0.48 |
|  | *R-*VK4-116 | 10 | 1.7 | ± | 0.34 | 1.5 | ± | 0.29 |
|  | Omeprazole | 50 | 20 | ± | 2.2 | 17 | ± | 1.9 |
| Lot 348B | 0.1% DMSO | 0 | 3.8 | ± | 0.23 |  | - |  |
|  | *R-*VK4-116 | 0.01 | 3.5 | ± | 0.31 | 0.92 | ± | 0.082 |
|  | *R-*VK4-116 | 0.1 | 3.5 | ± | 0.14 | 0.91 | ± | 0.037 |
|  | *R-*VK4-116 | 1 | 3.7 | ± | 0.31 | 0.99 | ± | 0.081 |
|  | *R-*VK4-116 | 3 | 4.1 | ± | 0.17 | 1.1 | ± | 0.046 |
|  | *R-*VK4-116 | 5 | 4.3 | ± | 0.33 | 1.1 | ± | 0.086 |
|  | *R-*VK4-116 | 10 | 4.2 | ± | 0.35 | 1.1 | ± | 0.091 |
|  | Omeprazole | 50 | 166 | ± | 4.8 | 44 | ± | 1.3 |
| Lot 399 | 0.1% DMSO | 0 | 8.2 | ± | 0.76 |  | - |  |
|  | *R-*VK4-116 | 0.01 | 8.8 | ± | 0.87 | 1.1 | ± | 0.11 |
|  | *R-*VK4-116 | 0.1 | 9.4 | ± | 0.75 | 1.1 | ± | 0.091 |
|  | *R-*VK4-116 | 1 | 9.3 | ± | 0.39 | 1.1 | ± | 0.047 |
|  | *R-*VK4-116 | 3 | 9.8 | ± | 1.1 | 1.2 | ± | 0.13 |
|  | *R-*VK4-116 | 5 | 10 | ± | 0.85 | 1.2 | ± | 0.10 |
|  | *R-*VK4-116 | 10 | 9.0 | ± | 0.18 | 1.1 | ± | 0.021 |
|  | Omeprazole | 50 | 186 | ± | 15 | 23 | ± | 1.8 |

Data are the mean ± SD from 3 wells except for * where n=2 due to an outlier.

^a^ Fold of vehicle control - the mean fold change of treated samples compared to vehicle control samples.

Table F. Effect of *R-*VK4-116 on CYP2B6 mRNA expression in human hepatocytes.

| **Hepatocyte Lot No.** | **Treatment** | **Concentration** | **Fold Inductiona** | | |
| --- | --- | --- | --- | --- | --- |
|  |  | **[µM]** |  |  |  |
| Lot 336 | 0.1% DMSO | 0 | 1.0 | ± | 0.24 |
|  | *R-*VK4-116 | 0.01 | 1.0 | ± | 0.33 |
|  | *R-*VK4-116 | 0.1 | 1.0 | ± | 0.19 |
|  | *R-*VK4-116 | 1 | 1.3 | ± | 0.17 |
|  | *R-*VK4-116 | 3 | 1.9 | ± | 0.24 |
|  | *R-*VK4-116 | 5 | 1.8 | ± | 0.38 |
|  | *R-*VK4-116 | 10 | 1.9 | ± | 0.26 |
|  | 0.1% DMSO | 0 | 1.0 | ± | 0.20 |
|  | Phenobarbital | 1000 | 11 | ± | 1.1 |
| Lot 348B | 0.1% DMSO | 0 | 1.0 | ± | 0.17 |
|  | *R-*VK4-116 | 0.01 | 0.98 | ± | 0.11 |
|  | *R-*VK4-116 | 0.1 | 1.2 | ± | 0.13 |
|  | *R-*VK4-116 | 1 | 1.0 | ± | 0.088 |
|  | *R-*VK4-116 | 3 | 1.1 | ± | 0.089 |
|  | *R-*VK4-116 | 5 | 1.1 | ± | 0.19 |
|  | *R-*VK4-116 | 10 | 1.1 | ± | 0.063 |
|  | 0.1% DMSO | 0 | 1.0 | ± | 0.080 |
|  | Phenobarbital | 1000 | 12 | ± | 1.8 |
| Lot 399 | 0.1% DMSO | 0 | 1.0 | ± | 0.15 |
|  | *R-*VK4-116 | 0.01 | 1.1 | ± | 0.19 |
|  | *R-*VK4-116 | 0.1 | 1.2 | ± | 0.064 |
|  | *R-*VK4-116 | 1 | 1.2 | ± | 0.24 |
|  | *R-*VK4-116 | 3 | 1.3 | ± | 0.20 |
|  | *R-*VK4-116 | 5 | 1.2 | ± | 0.36 |
|  | *R-*VK4-116 | 10 | 1.2 | ± | 0.11 |
|  | 0.1% DMSO | 0 | 1.0 | ± | 0.052 |
|  | Phenobarbital | 1000 | 11 | ± | 0.77 |

Data are the mean ± SD from 3 wells

^a^ Fold of vehicle control - the mean fold change of treated samples compared to vehicle control samples.

**Table G. Effect of *R-*VK4-116 on CYP2B6 induction activity in human hepatocytes**

| **Hepatocyte Lot No.** | **Treatment** | **[µM]** | **Bupropion Hydroxylation** | | | **Fold of Vehicle Controla** | | |
| --- | --- | --- | --- | --- | --- | --- | --- | --- |
|  |  |  | **pmol/min/106 cells** | | |  |  |  |
| Lot 336 | 0.1% DMSO | 0 | 0.92 | ± | 0.046 |  | - |  |
|  | *R-*VK4-116 | 0.01 | 1.1 | ± | 0.14 | 1.2 | ± | 0.15 |
|  | *R-*VK4-116 | 0.1 | 0.97 | ± | 0.070 | 1.1 | ± | 0.076 |
|  | *R-*VK4-116 | 1 | 1.0 | ± | 0.039 | 1.1 | ± | 0.043 |
|  | *R-*VK4-116 | 3 | 1.0 | ± | 0.040 | 1.1 | ± | 0.044 |
|  | *R-*VK4-116 | 5 | 0.96 | ± | 0.082 | 1.1 | ± | 0.089 |
|  | *R-*VK4-116 | 10 | 0.97 | ± | 0.12 | 1.1 | ± | 0.13 |
|  | Phenobarbital | 1000 | 3.6 | ± | 0.17 | 4.0 | ± | 0.19 |
| Lot 348B | 0.1% DMSO | 0 | 4.4 | ± | 0.011 |  | - |  |
|  | *R-*VK4-116 | 0.01 | 5.0 | ± | 0.34 | 1.1 | ± | 0.077 |
|  | *R-*VK4-116 | 0.1 | 4.5 | ± | 0.68 | 1.0 | ± | 0.15 |
|  | *R-*VK4-116 | 1 | 4.5 | ± | 0.10 | 1.0 | ± | 0.023 |
|  | *R-*VK4-116 | 3 | 4.1 | ± | 0.13 | 0.94 | ± | 0.030 |
|  | *R-*VK4-116 | 5 | 3.8 | ± | 0.23 | 0.85 | ± | 0.051 |
|  | *R-*VK4-116 | 10 | 3.9 | ± | 0.37 | 0.89 | ± | 0.085 |
|  | Phenobarbital | 1000 | 38 | ± | 4.6 | 8.6 | ± | 1.1 |
| Lot 399 | 0.1% DMSO | 0 | 2.0 | ± | 0.16 |  | - |  |
|  | *R-*VK4-116 | 0.01 | 2.0 | ± | 0.052 | 1.0 | ± | 0.026 |
|  | *R-*VK4-116 | 0.1 | 2.0 | ± | 0.026 | 1.0 | ± | 0.013 |
|  | *R-*VK4-116 | 1 | 2.1 | ± | 0.068 | 1.1 | ± | 0.034 |
|  | *R-*VK4-116 | 3 | 2.3 | ± | 0.14 | 1.2 | ± | 0.069 |
|  | *R-*VK4-116 | 5 | 2.2 | ± | 0.17 | 1.1 | ± | 0.084 |
|  | *R-*VK4-116 | 10 | 2.1 | ± | 0.31 | 1.1 | ± | 0.16 |
|  | Phenobarbital | 1000 | 38 | ± | 2.4 | 19 | ± | 1.2 |

Data are the mean ± SD from 3 wells.

^a^ Fold of vehicle control - the mean fold change of treated samples compared to vehicle control samples.

**Table H. Effect of *R-*VK4-116 on CYP3A4 induction activity in human hepatocytes**

| **Hepatocyte Lot No.** | **Treatment** | **[µM]** | **Testosterone-6β- Hydroxylation** | | | **Fold of Vehicle Controla** | | |
| --- | --- | --- | --- | --- | --- | --- | --- | --- |
|  |  |  | **pmol/min/106 cells** | | |  |  |  |
| Lot 336 | 0.1% DMSO | 0 | 9.4 | ± | 0.45 |  | - |  |
|  | *R-*VK4-116 | 0.01 | 9.1 | ± | 0.63 | 0.96 | ± | 0.067 |
|  | *R-*VK4-116 | 0.1 | 7.5 | ± | 1.1 | 0.80 | ± | 0.12 |
|  | *R-*VK4-116 | 1 | 6.3 | ± | 0.18 | 0.66 | ± | 0.019 |
|  | *R-*VK4-116 | 3 | 5.9 | ± | 0.69 | 0.63 | ± | 0.073 |
|  | *R-*VK4-116 | 5 | 6.1 | ± | 0.35 | 0.64 | ± | 0.037 |
|  | *R-*VK4-116 | 10 | 5.2 | ± | 0.60 | 0.55 | ± | 0.064 |
|  | Rifampicin | 10 | 86 | ± | 6.4 | 9.1 | ± | 0.68 |
| Lot 348B | 0.1% DMSO | 0 | 91 | ± | 5.9 |  | - |  |
|  | *R-*VK4-116 | 0.01 | 95 | ± | 9.2 | 1.0 | ± | 0.10 |
|  | *R-*VK4-116 | 0.1 | 90 | ± | 3.3 | 1.0 | ± | 0.036 |
|  | *R-*VK4-116 | 1 | 63 | ± | 5.7 | 0.70 | ± | 0.063 |
|  | *R-*VK4-116 | 3 | 50 | ± | 3.0 | 0.56 | ± | 0.033 |
|  | *R-*VK4-116 | 5 | 50 | ± | 1.4 | 0.56 | ± | 0.016 |
|  | *R-*VK4-116 | 10 | 44 | ± | 3.3 | 0.49 | ± | 0.037 |
|  | Rifampicin | 10 | 320 | ± | 22 | 3.5 | ± | 0.24 |
| Lot 399 | 0.1% DMSO | 0 | 55 | ± | 2.4 |  | - |  |
|  | *R-*VK4-116 | 0.01 | 58 | ± | 1.6 | 1.0 | ± | 0.028 |
|  | *R-*VK4-116 | 0.1 | 49 | ± | 8.7 | 0.89 | ± | 0.16 |
|  | *R-*VK4-116 | 1 | 25 | ± | 1.1 | 0.45 | ± | 0.020 |
|  | *R-*VK4-116 | 3 | 18 | ± | 0.93 | 0.32 | ± | 0.017 |
|  | *R-*VK4-116 | 5 | 16 | ± | 0.83 | 0.30 | ± | 0.015 |
|  | *R-*VK4-116 | 10 | 13 | ± | 0.70 | 0.23 | ± | 0.013 |
|  | Rifampicin | 10 | 474 | ± | 25 | 8.6 | ± | 0.44 |

Data are the mean ± SD from 3 wells.

^a^ Fold of vehicle control - the mean fold change of treated samples compared to vehicle control samples.

**CYP Inhibition Assay**

**Table I. Effect of *R-*VK4-116 on CYP inhibition activity in human liver microsomes.**

^a^ Results are presented as Mean ± SD of three replicates.

* Compound was not completely soluble at this concentration.

| **Test article/**  **Control Inhibitor** | **Conc (μM)** | **CYP activity (% of Control)^a^** | | | | | | | |
| --- | --- | --- | --- | --- | --- | --- | --- | --- | --- |
|  |  | **1A2**  **(Phenacetin)** | **2B6**  **(Bupropion)** | **2C8**  **(Paclitaxel)** | **2C9**  **(Diclofenac)** | **2C19**  **(Mephenytoin)** | **2D6**  **(Bufuralol)** | **3A4**  **(Testosterone)** | **3A4**  **(Midazolam)** |
| ***R-*VK4-116** | **0.1** | 93.7 ± 5.5 | 99.6 ± 7.5 | 85.9 ± 18.2 | 89.5 ± 9.2 | 101.8 ± 4.6 | 92.6 ± 9.0 | 96.5 ± 3.6 | 97.5 ± 3.9 |
|  | **0.3** | 87.7 ± 2.4 | 90.9 ± 2.5 | 68.2 ± 2.8 | 79.0 ± 2.7 | 96.2 ± 5.0 | 86.1 ± 4.0 | 93.2 ± 3.8 | 93.9 ± 2.4 |
|  | **1** | 89.9 ± 0.9 | 91.2 ± 1.4 | 70.5 ± 7.1 | 80.9 ± 2.2 | 97.3 ± 2.2 | 84.3 ± 3.7 | 94.2 ± 2.4 | 95.3 ± 0.9 |
|  | **3** | 95.7 ± 5.2 | 99.8 ± 7.6 | 95.7 ± 11.2 | 92.3 ± 10.1 | 95.9 ± 3.6 | 92.1 ± 6.8 | 92.8 ± 4.8 | 95.6 ± 4.2 |
|  | **10** | 96.3 ± 4.7 | 97.5 ± 6.6 | 89.8 ± 19.7 | 91.5 ± 7.3 | 99.6 ± 3.3 | 94.7 ± 6.5 | 93.9 ± 2.1 | 96.9 ± 2.0 |
|  | **30*** | 88.7 ± 1.9 | 89.3 ± 1.2 | 75.8 ± 4.5 | 80.9 ± 0.9 | 96.7 ± 3.5 | 83.1 ± 1.6 | 94.2 ± 1.5 | 92.2 ± 0.7 |
|  | **60*** | 88.2 ± 2.3 | 91.0 ± 0.7 | 75.3 ± 6.4 | 81.8 ± 3.1 | 95.0 ± 5.4 | 84.5 ± 1.8 | 92.1 ± 1.1 | 92.6 ± 1.0 |
|  | **100*** | 94.0 ± 5.2 | 97.0 ± 4.9 | 80.6 ± 15.2 | 93.4 ± 8.1 | 99.7 ± 3.4 | 95.0 ± 7.5 | 95.5 ± 3.7 | 96.9 ± 3.2 |
| **Furafylline (1A2)** | **10** | 49.0 ± 5.3 | 97.3 ± 2.9 | 89.9 ± 1.4 | 96.3 ± 1.4 | 94.3 ± 8.1 | 94.7 ± 1.1 | 95.1 ± 5.6 | 97.2 ± 4.7 |
| **ThioTEPA (2B6)** | **10** | 93.8 ± 4.3 | 52.0 ± 0.1 | 96.8 ± 4.4 | 92.9 ± 1.5 | 108.1 ± 4.3 | 90.7 ± 2.0 | 91.4 ± 1.3 | 92.6 ± 2.2 |
| **Montelukast (2C8)** | **10** | 91.0 ± 1.8 | 90.5 ± 1.3 | 10.3 ± 0.7 | 75.4 ± 0.9 | 105.0 ± 2.0 | 88.4 ± 1.8 | 93.1 ± 2.0 | 93.8 ± 3.9 |
| **Sulfaphenazole (2C9)** | **3** | 98.1 ± 10.8 | 100.2 ± 5.8 | 97.6 ± 19.2 | 23.6 ± 3.3 | 102.4 ± 6.5 | 94.8 ± 7.2 | 95.9 ± 4.3 | 98.7 ± 5.2 |
| **Nootkatone (2C19)** | **30** | 91.3 ± 4.8 | 23.8 ± 2.7 | 78.8 ± 12.5 | 84.6 ± 8.5 | 57.3 ± 5.7 | 91.9 ± 8.4 | 79.4 ± 5.4 | 84.5 ± 4.0 |
| **Quinidine (2D6)** | **2** | 88.3 ± 1.0 | 91.6 ± 2.4 | 71.3 ± 2.2 | 82.7 ± 1.1 | 97.7 ± 2.3 | 17.3 ± 0.2 | 93.2 ± 1.7 | 91.7 ± 0.8 |
| **Ketoconazole (3A4)** | **5** | 70.7 ± 2.2 | 49.1 ± 0.7 | 45.7 ± 4.7 | 65.8 ± 1.9 | 82.9 ± 2.8 | 77.6 ± 4.1 | 2.7 ± 0.1 | 5.1 ± 0.2 |

**Transporter-Mediated Substrate Assay**

*R-*VK4-116 was tested as a potential substrate or inhibitor for human OAT1, OAT3, OCT2, OATP1B1, OATP1B3, MATE1, and MATE2-K -mediated transport in the presence of 0.1% BSA.

At 1 µM of *R-*VK4-116, less than a 2-fold difference in uptake was observed in transporter-transfected cells compared to control cells for each SLC transporter studied. Therefore, *R-*VK4-116 does not appear to be a substrate as defined by regulatory guidance documents for human OAT1, OAT3, OCT2, OATP1B1, OATP1B3, MATE1, or MATE2-K under the study conditions **(Figure 1).**


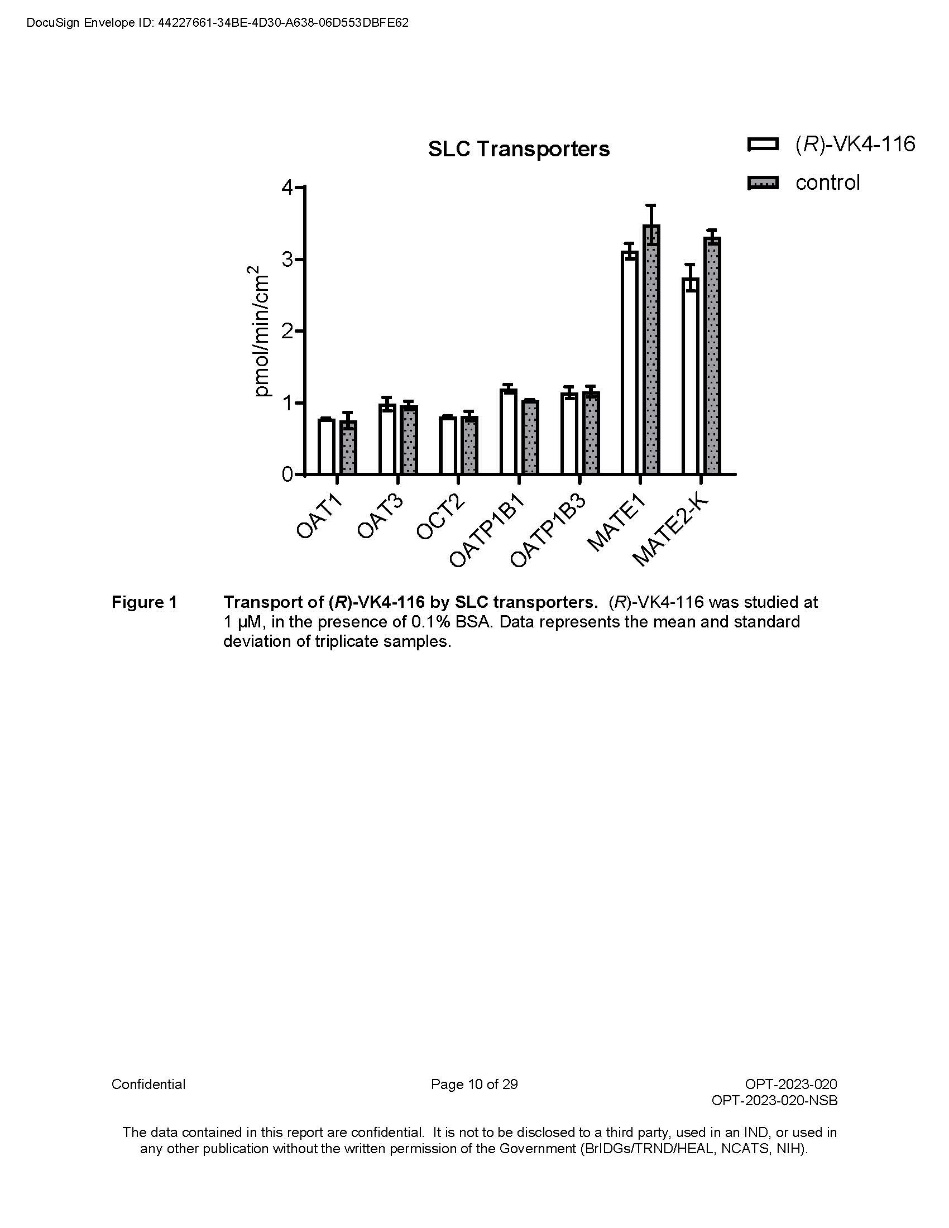


The limit of quantitation was 1 nM *R-*VK4-116, corresponding to Papp values of < 0.1 x 10^-6^ cm/sec for B->A transport and < 0.2 x 10^-6^ cm/sec for A->B transport. As a result, it was not possible to calculate efflux ratios or determine whether *R-*VK4-116 was a substrate for either BCRP or P-gp under these conditions (Table IV).

**Table J. *In vitro* data for the transport of *R-*VK4-116 mediated by human BCRP and P-gp.**

| **Transporter** | **Test Conditions** | **P_app_ (x10^-6^ cm/s)** | | **Vectorial Net Transport**  **(B->A) - (A->B)**  **(pmoles/min/cm^2^)** | **Efflux Ratio** |
| --- | --- | --- | --- | --- | --- |
|  |  | **(B->A)** | **(A->B)** |  |  |
| **BCRP** | 2 µM prazosin | 47.7 ± 2.77 | 8.06 ± 0.76 | 4.15 ± 0.29 | 5.92 ± 0.34 |
|  | 1 µM *R-*VK4-116 | < 0.1 | < 0.2 | ND | ND |
| **P-gp** | 0.1 µM quinidine | 80.7 ± 4.23 | 2.62 ± 0.42 | 0.52 ± 0.03 | 30.8 ± 1.62 |
|  | 1 µM *R-*VK4-116 | < 0.1 | < 0.2 | ND | ND |

Lysosomal Perturbation Potential of *R-*VK4-116.

Figure 2. Fluorescence fold change (red lysosomal stain and blue nuclear stain) calculated for HepG2 cells treated for 24 (Day 1), 48 (Day 2) and 72 hr (Day 3) with different concentrations of Verapamil (A), Piroxicam (B) and *R-*VK4-116 (C).

**MT Assay:**

**Figure 3.** The MT assay panel results following 2-week treatment of HepG2 cells with 100 µM zalcitabine (positive control). A ~80% decrease in OXPHOS 1, ~20% decrease in OXPHOS 3, and ~70% decrease in OXPHOS 4 levels in HepG2 cells was observed (Figure 3A), while a ~35% increase in total glutathione content following two weeks of treatment with zalcitabine compared with the solvent control (Figure 3B). No significant decrease in OXPHOS 5 and total ATP levels or increase in caspase 3 or ROS/RNS levels in HepG2 cells was observed.

**Enzyme and Uptake Assays**

**Figure 4:** *R-*VK4-116
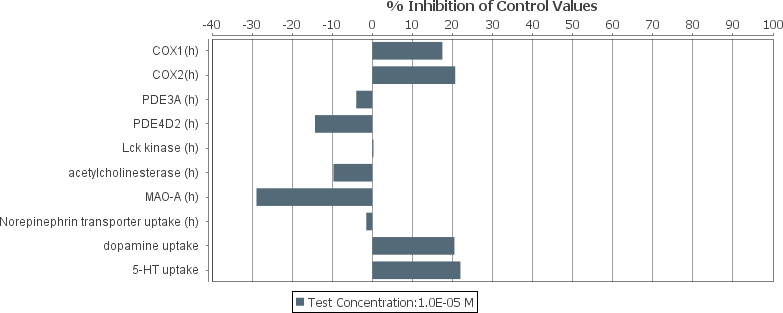
 was tested at 10 µM for enzyme and uptake assays.

Test concentration: *R-*VK4-116 (10 µM)

**Cellular and Nuclear Receptor Functional Assays**


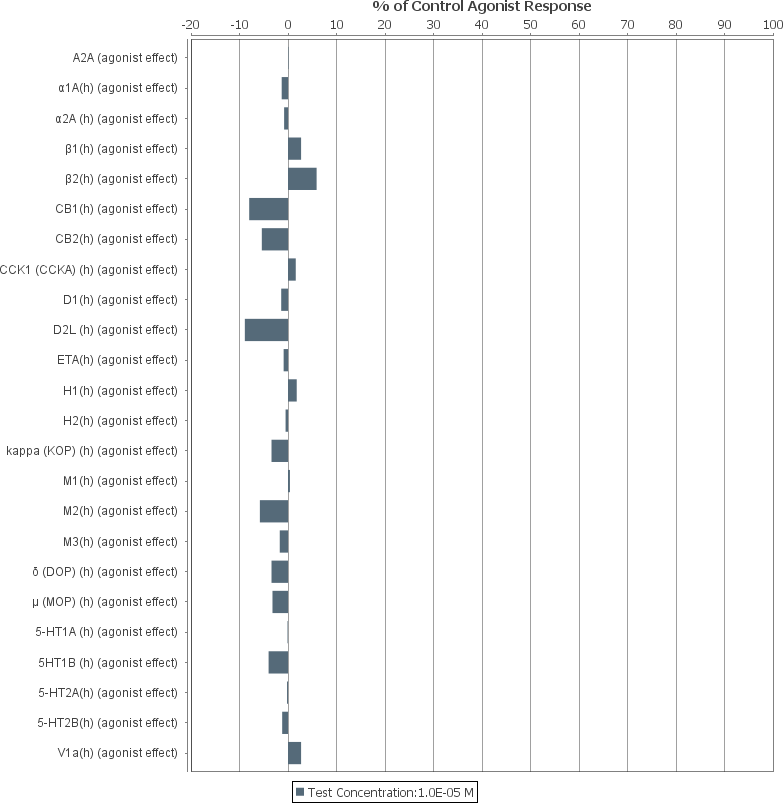
**Figure 5:** Agonist effect of *R-*VK4-116 (10 µM)


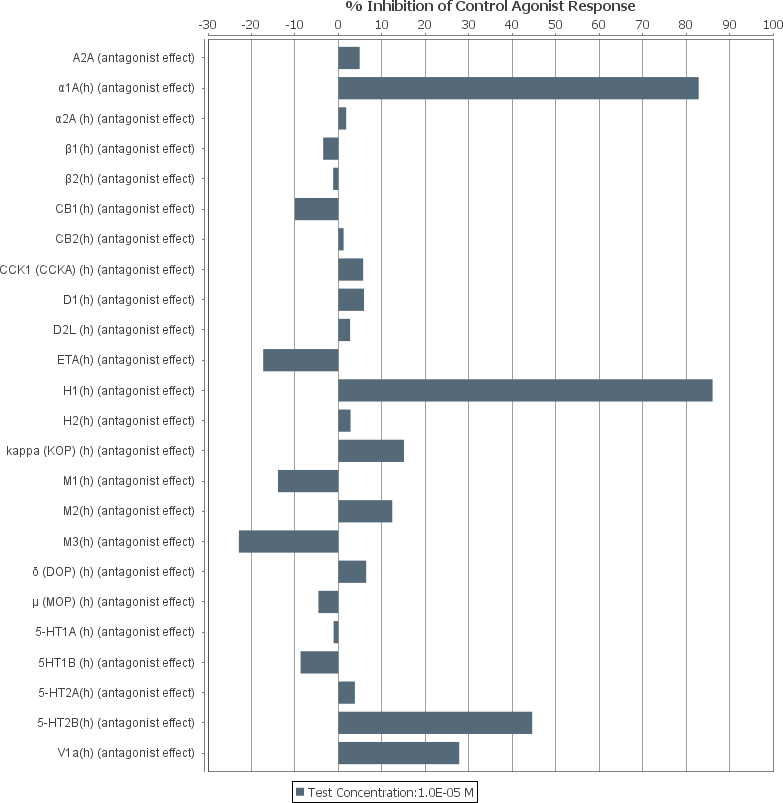
**Figure 6:** Antagonist effect of *R-*VK4-116 (10 µM)

**Table K. Cardiac panel with % inhibition (Mean of two sample results)**

| **Name of compound** | **Concentration (µM)** | **Mean % inhibition on hERG** | **Mean % inhibition on hKir2.1**  **(Peak)** | **Mean % inhibition on hKir2.1**  **(End)** |
| --- | --- | --- | --- | --- |
| *(R)*-VK4-116 | 0.01 | -3.97 | 9.54 | 8.24 |
| *(R)*-VK4-116 | 0.1 | -4.66 | 14.42 | 12.22 |
| *(R)*-VK4-116 | 1 | 17.70 | 19.01 | 16.36 |
| *(R)*-VK4-116 | 10 | 54.60 | 21.58 | 18.23 |
| *(R)*-VK4-116 | 100 | 82.26 | 52.64 | 97.19 |
| 0.3% DMSO (Vehicle) |  | -2.84 | 4.93 | -0.22 |
| 0.3% DMSO (Vehicle) |  | -2.39 | 7.56 | 1.00 |
| 0.3% DMSO (Vehicle) |  | -1.74 | 3.31 | 4.25 |
| 0.3% DMSO (Vehicle) |  | 1.13 | 11.36 | 17.01 |
| 0.3% DMSO (Vehicle) |  | 2.99 | 8.96 | 12.40 |
| E-4031 (Positive control) | 0.03 | 7.60 |  |  |
| E-4031 (Positive control) | 0.3 | 19.38 |  |  |
| E-4031 (Positive control) | 3 | 43.08 |  |  |
| E-4031 (Positive control) | 30 | 81.66 |  |  |
| E-4031 (Positive control) | 300 | 97.21 |  |  |
| Barium Chloride (Positive control) | 1 |  | 6.70 | 11.87 |
| Barium Chloride (Positive control) | 3 |  | 11.92 | 30.43 |
| Barium Chloride (Positive control) | 10 |  | 17.51 | 55.57 |
| Barium Chloride (Positive control) | 30 |  | 27.21 | 85.21 |
| Barium Chloride (Positive control) | 100 |  | 45.99 | 89.33 |

**Tale L. S-score and binding constants (Kds)**

| **Tested compound** | **Screening concentration** | **S-score with % Control** | **Gene (S-score with % control)** | **Selectivity score** | **Binding constant** |
| --- | --- | --- | --- | --- | --- |
| *R*-VK4-116 | 1 µM | <35 | CHEK2 (19) | 0.012 | >10 µM |
|  |  |  | HPK1 (29) | 0.012 | >10 µM |
|  |  |  | MARK3 (0) | 0.012 | >10 µM |
|  |  |  | SRPK2 (21) | 0.012 | >10 µM |
|  |  |  | TNK1 (17) | 0.012 | >10 µM |
